# Vimentin is required for tumor progression and metastasis in a mouse model of non–small cell lung cancer

**DOI:** 10.1038/s41388-023-02703-9

**Published:** 2023-05-09

**Authors:** Alexandra L. Berr, Kristin Wiese, Gimena dos Santos, Clarissa M. Koch, Kishore R. Anekalla, Martha Kidd, Jennifer M. Davis, Yuan Cheng, Yuan-Shih Hu, Karen M. Ridge

**Affiliations:** 1 Department of Biomedical Engineering, Northwestern University, Chicago, IL USA; 2 Division of Pulmonary and Critical Care Medicine, Northwestern University, Chicago, IL USA; 3 Department of Cell and Molecular Biology, Northwestern University, Chicago, IL USA

**Keywords:** Non-small-cell lung cancer, Intermediate filaments, Cell death

## Abstract

Vimentin is highly expressed in metastatic cancers, and its expression correlates with poor patient prognoses. However, no causal in vivo studies linking vimentin and non–small cell lung cancer (NSCLC) progression existed until now. We use three complementary in vivo models to show that vimentin is required for the progression of NSCLC. First, we crossed *LSL-Kras*^G12D^; *Tp53*^fl/fl^ mice (*KPV*^+/+^) with vimentin knockout mice (*KPV*^−/−^) to demonstrate that *KPV*^−/−^ mice have attenuated tumor growth and improved survival compared with *KPV*^+/+^ mice. Next, we therapeutically treated *KPV*^+/+^ mice with withaferin A (WFA), an agent that disrupts vimentin intermediate filaments (IFs). We show that WFA suppresses tumor growth and reduces tumor burden in the lung. Finally, luciferase-expressing *KPV*^+/+^, *KPV*^−/−^, or *KPV*^Y117L^ cells were implanted into the flanks of athymic mice to track cancer metastasis to the lung. In *KPV*^Y117L^ cells, vimentin forms oligomers called unit-length filaments but cannot assemble into mature vimentin IFs. *KPV*^–/–^ and *KPV*^Y117L^ cells fail to metastasize, suggesting that cell-autonomous metastasis requires mature vimentin IFs. Integrative metabolomic and transcriptomic analysis reveals that *KPV*^–/–^ cells upregulate genes associated with ferroptosis, an iron-dependent form of regulated cell death. *KPV*^–/–^ cells have reduced glutathione peroxidase 4 (GPX4) levels, resulting in the accumulation of toxic lipid peroxides and increased ferroptosis. Together, our results demonstrate that vimentin is required for rapid tumor growth, metastasis, and protection from ferroptosis in NSCLC.

## Introduction

Lung adenocarcinoma (LUAD) is a type of non–small cell lung cancer (NSCLC) and is the most common subtype of lung cancer, representing 40% of all lung cancer diagnoses [1]. Vimentin, a type III intermediate filament, is highly expressed in lung cancer and is associated with metastatic tissue and lower survival rates in patients with NSCLC [2–4]. Despite this association, a causal, in vivo link between vimentin and disease progression has not been established.

Vimentin’s role in the metastatic process has been extensively investigated in vitro. During epithelial-to-mesenchymal transition (EMT), epithelial cells lose their apicobasal polarity and intercellular adhesion properties by downregulating epithelial cell-associated genes, including E-cadherin and cytokeratins, and progressively acquire migratory and invasive capabilities associated with the mesenchymal cell phenotype by upregulating genes, such as N-cadherin and vimentin [5, 6]. EMT-mediated tumor metastasis is stimulated by transcription factors, such as TWIST1, SNAI1, and ZEB1, known to upregulate vimentin [7–10]. Formation and elongation of the invadopodia, a proteolytically active plasma membrane projection that facilitates cancer cell invasion across the basement membrane and migration through the collagen-rich interstitial space, requires vimentin [11]. Vimentin is crucial for establishing front-rear polarity, which is necessary for the efficient migration of tumor cells [12]. Previous studies identified these vimentin-dependent mechanisms in cancer cell invasive migration, metastasis, and poor prognosis in patients with lung, breast, head and neck, and bone cancer cells [9, 13–21].

Tumors must maintain redox homeostasis to survive and grow. An emerging mechanism by which tumor cells manage oxidative stress is ferroptosis. Ferroptosis is a form of iron-dependent regulated cell death (RCD) initiated by the peroxidization of phospholipids (PLs) containing polyunsaturated fatty acid chains (PUFA-PLs). Extrinsic and intrinsic pathways regulate ferroptosis [22]. The extrinsic pathway regulates ferroptosis by inhibiting cell membrane transporters, such as the system Xc− cystine/glutamate transporter (consisting of SLC7A11 and SLC3A2 subunits) or through iron uptake via transferrin and lactotransferrin transporters. The intrinsic pathway is activated by blocking intracellular antioxidant enzymes, such as glutathione peroxidase 4 (GPX4). Glutathione is a cofactor for GPX4, which neutralizes lipid hydroperoxides and protects the cell from ferroptosis. KRAS-mutant LUAD cells express high levels of SLC7A11 and GPX4; pharmacological or genetic inhibition of either SLC7A11 and GPX4 attenuates tumor growth in vivo [23, 24]. Of note, the regulation of ferroptosis is not entirely understood.

While prior studies established a role for vimentin in cancer cells, these experiments relied on either cell lines treated with exogenous agents to suppress vimentin expression or cells derived from the vimentin knockout (*Vim*^*−/−*^) mice that lacked oncogene activation. In the present study, we used the clinically relevant *LSL*-*Kras*^G12D^; *Tp53*^fl/fl^ (*KPV*^+/+^) genetically engineered mouse model (GEMM), which reliably recapitulates human NSCLC in pathology, disease progression, clinical outcome, and response to therapies [25]. To identify the role of vimentin in LUAD, we crossed the *LSL*-*Kras*^G12D^; *Tp53*^fl/fl^ with *Vim*^*−/−*^ mice, thereby creating *KPV*^−/−^ mice [26]. We show that *KPV*^−/−^ mice have reduced lung tumor burden and increased survival rates compared with *KPV*^+/+^ mice. Finally, we use an allograft tumor model to demonstrate that *KPV*^−/−^ cells cannot metastasize to the lung. Compared with *KPV*^+/+^ cells, *KPV*^−/−^ cells display impaired EMT and increased susceptibility to ferroptosis. Together, our results show that an intact vimentin intermediate filament network is required for the rapid progression and metastasis of LUAD.

## Results

### Generation of an *LSL-Kras*^G12D/+^**;*Tp53***^**fl/fl**^; *Vim*^−/−^ (*KPV*^−/−^) genetically engineered mouse model

In patients with LUAD, 30% have activating mutations in the *KRAS* proto-oncogene, and 60% have inactivating mutations in the tumor suppressor gene *TP53* [27–30]. Therefore, we used the *LSL-Kras*^G12D/+^; *Tp53*^fl/fl^ (*KPV*^+/+^) GEMM to recapitulate the tumorigenesis and metastasis observed in LUAD [25]. When adenoviral Cre recombinase (Ad-Cre) is delivered intratracheally to *KPV*^+/+^ mice, tumors develop as early as 2 weeks post-infection (w.p.i.) [25], with ~50% of mice developing metastatic lesions in the mediastinal lymph nodes and the pleural spaces of the thoracic cavity [31]. We crossed the *Vimentin*^−/−^ mouse to the *LSL-Kras*^G12D/+^; *Tp53*^fl/fl^ mouse to create the *KPV*^−/−^ mouse [26] (Supplementary Fig. S1A). This novel *KPV*^−/−^ mouse lacks vimentin expression throughout the lungs (Supplementary Fig. S1B). *Rosa26-LSL-LacZ* reporter mice were used to validate the intratracheal delivery of Ad-Cre [32]. Mice infected with Ad-Cre demonstrated homogenous, positive lacZ expression, while mice treated with the adenoviral null construct (Ad-null) did not express lacZ (Supplementary Fig. S1C). Ad-Cre was administered intratracheally, and disruption of the *Kras* allele and accumulation of mutant KRAS protein was validated by PCR and Western blot, respectively (Supplementary Fig. S1D, E). Similarly, p53 deletion was confirmed by qPCR and Western blot (Supplementary Fig. S1F, G). These results demonstrate the utility of a novel *KPV*^−/−^ GEMM to define a causal role of vimentin in lung adenocarcinoma metastasis.

### Vimentin deficiency increases survival and reduces tumor burden

**Fig. 1:**
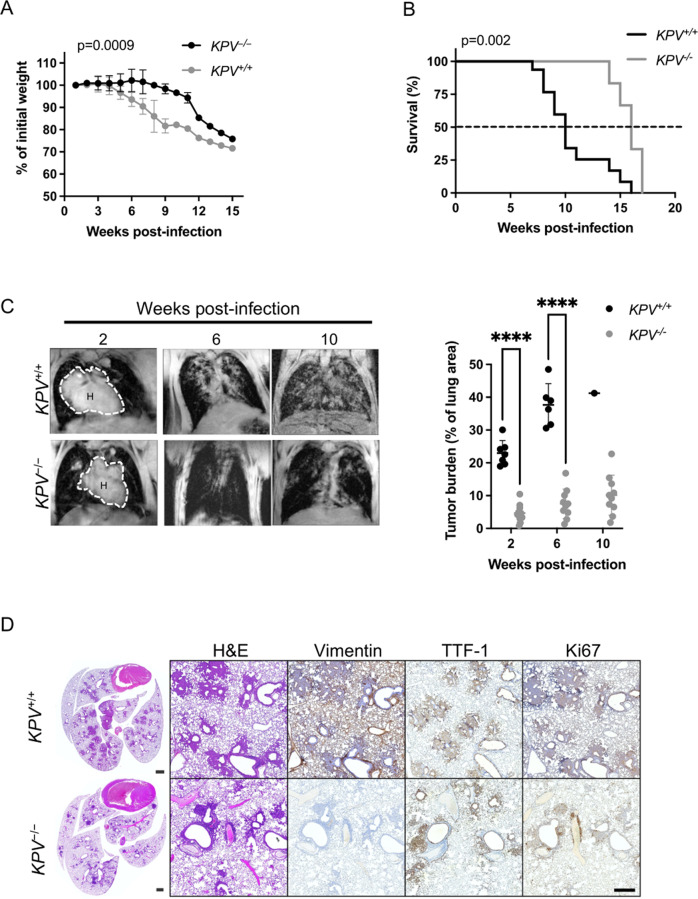
Vimentin-null mice have reduced tumor burden and improved survival in a preclinical *LSL-Kras*^G12D^*Tp53*^fl/fl^-driven mouse model of lung cancer. *LSL*-*Kras*^G12D^*Tp53*^fl/fl^ (*KPV*^+/+^) mice were crossed with *Vim*^*−/−*^ mice to produce *KPV*^−/−^ mice, then *KPV*^+/+^ and *KPV*^−/−^ mice were intubated with 10^9^ PFUs of adenoviral Cre. **A** Weight loss (*n* = 6 mice for *KPV*^+/+^ group; *n* = 7 mice for KPV^−/−^ group; mixed model ANOVA, for *KPV*^+/+^ vs. *KPV*^−/−^, *p* = 0.0009) and **B** survival (*n* = 15 mice for *KPV*^+/+^ group; *n* = 10 mice for *KPV*^−/−^ group; Mantel-Cox log rank test, *p* = 0.002) were monitored. **C** Representative MRI scans (*left*) showing mouse lung tumors at 2, 6, and 10 weeks post-infection with 10^9^ PFUs of adenoviral Cre. Dotted lines and H indicate heart. Tumor burden was quantified using Jim software (*right*). Each point represents one mouse (^****^p < 0.0001 by unpaired, two-tailed *t*-test). **D** Lungs were isolated from *KPV*^+/+^ mice (6 weeks post-infection shown) and *KPV*^−/−^ mice (7 weeks post-infection shown) infected with 10^9^ PFUs of adenoviral Cre. Shown from left to right are representative fixed whole lung sections with H&E staining and close-up views of fixed lung sections with H&E staining and vimentin, TTF-1, and Ki67 immunohistochemical staining. Positively immunostained cells appear brown, and nuclei are dyed blue. Scale bars: 1 mm (whole lungs, *left*), 200 µm (*right*). This figure represents combined data from three independent experiments.

Cancer cachexia is a common manifestation of morbidity in human cancer patients and is associated with a poor prognosis in patients with advanced disease. *KPV*^+/+^ and *KPV*^−/−^ mice were administered Ad-Cre, and their weight was recorded weekly. *KPV*^+/+^ mice showed a rapid and profound decline in total body weight starting at 4 w.p.i., while *KPV*^−/−^ mice did not exhibit weight loss until 9 w.p.i., suggesting less advanced disease in the vimentin-deficient mice (Fig. 1A). *KPV*^−/−^ mice lived significantly longer than *KPV*^+/+^ mice, with a median survival of 15.5 w.p.i. compared to 10 w.p.i. in the *KPV*^+/+^ mice (Fig. 1B). Lung tumor development was assessed using magnetic resonance imaging (MRI). At 6 w.p.i., *KPV*^−/−^ mice had an average lung tumor burden of 7.5%, significantly lower than the 37% tumor burden observed in *KPV*^+/+^ mice (Fig. 1C, Supplementary Fig. S1H). Immunohistochemistry (IHC) staining for vimentin, TTF-1, and Ki67 was performed on serial sections of lung tissue from *KPV*^+/+^ and *KPV*^−/−^ mice at 6 w.p.i. *KPV*^+/+^ tumors and normal adjacent lung tissue expressed vimentin, but *KPV*^−/−^ lung tissue did not express vimentin (Fig. 1D). TTF-1 is a biomarker associated with LUAD [33]. *KPV*^−/−^ lungs had fewer TTF-1-positive cells than *KPV*^+/+^ lungs (Fig. 1D, Supplementary Fig. S1I). *KPV*^+/+^ and *KPV*^−/−^ lung tumors displayed similar levels of Ki67 staining by IHC (Fig. 1D, Supplementary Fig. S1I). However, *KPV*^+/+^ cells had increased proliferation rates, as quantitatively measured by BrdU incorporation, compared to *KPV*^−/−^ cells isolated from primary lung tumors 6 w.p.i. (Supplementary Fig. S1J). At 8 w.p.i., *KPV*^+/+^ mice displayed significantly more hyperplastic lesions than *KPV*^−/−^ mice (36 ± 5 vs. 14 ± 2, respectively). At 12 w.p.i., *KPV*^+/+^ mice displayed an increased number of adenomas and adenocarcinomas (6.3 ± 1.8 adenomas and 1.2 ± 0.2 adenocarcinomas per *KPV*^+/+^ mouse) compared to *KPV*^−/−^ mice (1.6 ± 1.0 adenomas and 0.04 ± 0.02 adenocarcinomas per *KPV*^−/−^ mouse) (Supplementary Fig. S1K). Additionally, mutant *Kras* transcripts were detected in liver tissue from *KPV*^+/+^ mice but not *KPV*^−/−^ mice, suggesting that vimentin-expressing cells form metastatic lesions (Supplementary Fig. S1D). Together, these data indicate that loss of vimentin suppresses tumor development in a murine model of LUAD, contributing to prolonged survival. In agreement with these findings, vimentin protein expression increased with tumor grade in human LUAD sections and corresponding lymph node sections containing LUAD metastatic lesions (Supplementary Fig. S1L).

### Transcriptional profiling reveals a less differentiated cancer phenotype in vimentin-null cancer cells

**Fig. 2:**
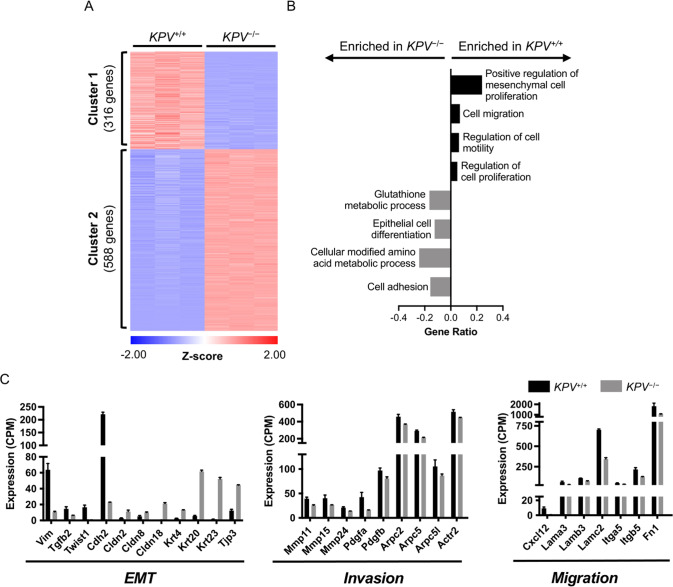
*KPV*^−/−^ cells have decreased expression of genes involved in EMT. Messenger RNA collected from *KPV*^+/+^ and *KPV*^−/−^ cells was subjected to RNA sequencing. **A** Differentially expressed genes (DEGs) between *KPV*^+/+^ and *KPV*^−/−^ cells were clustered using K-means clustering. **B** Genes enriched in Cluster 1 (316 genes) and Cluster 2 (588 genes) were subjected to GO enrichment analysis. GO Processes with FDR < 0.05 are shown. GeneRatio is the number of genes in the cluster associated with the GO process divided by the total number of genes in that GO process. **C** Expression values (counts per million; CPM) of select genes are shown. *N* = 3 for each group. Data in panel **C** are presented as the mean ± standard deviation. All gene comparisons (*KPV*^+/+^ vs. *KPV*^−/−^) have FDR < 0.05 after adjusting for multiple comparisons; therefore, all gene differences shown between *KPV*^+/+^ vs. *KPV*^−/−^ cells are statistically significant.

To better understand how vimentin is involved in the molecular pathways of LUAD, we used RNA sequencing (RNA-seq) to identify genes that have altered expression in the absence of vimentin. Epithelial-derived cancer cells (CD45-negative, EpCAM-positive) were isolated from *KPV*^+/+^ and *KPV*^−/−^ lungs at 6 w.p.i. with Ad-Cre (hereafter referred to as *KPV*^+/+^ and *KPV*^−/−^ cells). Western blot and immunofluorescence staining confirmed the absence of vimentin in *KPV*^−/−^ cells (Supplementary Fig. S2A, B). We isolated messenger RNA (mRNA) from *KPV*^+/+^ and *KPV*^−/−^ cells and performed RNA-seq. *KPV*^+/+^ and *KPV*^−/−^ cells clustered together by genotype via principal component analysis (PCA). Loss of vimentin in *KPV*^−/−^ cells was the main driver of sample variance (PC1: 97.6%) (Supplementary Fig. S2C), and Pearson’s correlation revealed minimal variance in intragroup replicates (Supplementary Fig. S2D). We identified 904 differentially expressed genes (DEGs) between the *KPV*^+/+^ and *KPV*^−/−^ cells (Supplementary Fig. S2E). K-means clustering identified two clusters: Cluster 1 contained 316 upregulated genes while Cluster 2 had 588 downregulated genes in *KPV*^+/+^ cells compared to *KPV*^−/−^ cells (Fig. 2A). Cluster 1 was enriched for genes associated with Gene Ontology (GO) processes *mesenchymal cell proliferation* and *cell migration*. In contrast, Cluster 2 was enriched for genes related to *metabolic processes, epithelial cell differentiation*, and *cell adhesion* (Fig. 2B). By exploring the DEGs that contribute to *mesenchymal cell proliferation* and *epithelial cell differentiation*, we found several EMT-associated genes upregulated in *KPV*^+/+^ cells, including *Vimentin, Twist1*, and *Cdh2*, the gene that codes for N-cadherin (Fig. 2C). This finding was confirmed by IHC of tumors and Western blots on isolated tumor cells, which showed an increase in N-cadherin in *KPV*^+/+^ cells compared to *KPV*^−/−^ cells (Supplementary Fig. S2A, F). In contrast, genes associated with epithelial cell phenotype, including claudins *Cldn2, Cldn8*, and *Cldn18*, and the epithelial marker *Epcam* and the surfactant protein *Sftpd*, were upregulated in *KPV*^−/−^ cells. These data suggest that *KPV*^−/−^ cells, but not *KPV*^+/+^ cells, fail to upregulate essential mesenchymal genes that confer metastatic potential.

We then explored the DEGs that contribute to the GO processes *cell motility* and *cell migration*, processes enriched in *KPV*^+/+^ cells. Cancer cells must form invadopodia, a process that relies on vimentin, to cross the basement membrane [11]. Accordingly, invadopodia-associated genes (*Arpc2, Arpc5, Arpc5l*, and *Actr2*) are significantly downregulated in *KPV*^−/−^ cells but upregulated in *KPV*^+/+^ cells (Fig. 2C). Invasion is potentiated by matrix metalloproteases (MMPs), which break down the basement membrane, allowing cells to move toward adjacent capillaries, a critical process in the metastatic cascade. *Mmp11, Mmp15*, and *Mmp24* are significantly upregulated in *KPV*^+/+^ cells but not in the *KPV*^−/−^ cells (Fig. 2C). Cells must then migrate across a collagen-rich interstitial space to reach the bloodstream. This process is coordinated by chemokines (*Cxcl12*), integrins (*Itga5* and *Itgb5*), and alterations in the ECM (*Lama3, Lamb3, Lamc2*, and *Fn1*); these genes are significantly downregulated in *KPV*^−/−^ cells compared to *KPV*^+/+^ cells (Fig. 2C). Together, these results suggest that vimentin is involved in regulating the early cellular events associated with the metastatic cascade.

### An intact vimentin network is required for cancer cell migration and invasion

**Fig. 3:**
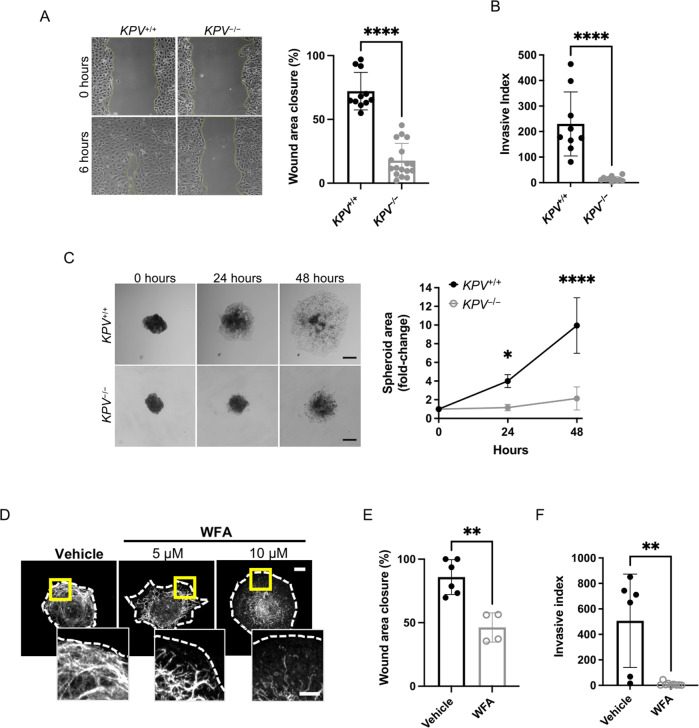
Vimentin is required for in vitro cancer cell migration and invasion. **A** A scratch wound assay was used to evaluate cell migration. Representative images are shown at 0 and 6 h following scratch formation. Wound area closure was compared to the starting value and quantified for *KPV*^+/+^ (*n* = 11) and *KPV*^−/−^ (*n* = 17) cells; each point represents a separate scratch wound. **B** Cell invasion through a Matrigel-coated transwell was measured over 48 h. Invasive index is the mean number of cells invaded per 20× magnification imaging field. *KPV*^+/+^ (*n* = 9) and *KPV*^−/−^ (*n* = 11) cell invasion data are plotted so that each point represents data from a single transwell assay. **C** *KPV*^+/+^ and *KPV*^−/−^ spheroids were suspended in type I collagen and spheroid growth was tracked over 48 h. Spheroid area was quantified relative to the initial area of each spheroid (*n* = 4 independent experiments). Scale bar: 200 µM. **D** *KPV*^+/+^ cells were treated with withaferin A (WFA; 5 or 10 µM) or DMSO vehicle control for 6 h. Cells were stained for vimentin (white; dotted line represents cell outline). Scale bar: 10 µm; inset: 5 µm. **E** *KPV*^+/+^ cells were treated with vehicle (*n* = 6) or 5 µM WFA (*n* = 4) and were subjected to a scratch wound assay. Wound area was quantified at 6 h. **F** KPV^+/+^ cells were plated atop a Matrigel-coated transwell and were treated with vehicle control (*n* = 6) or 5 µM WFA (*n* = 9); invasion was quantified at 48 h via invasive index as described above. Data are presented as the mean ± standard deviation. The *p*-values were calculated using an unpaired, two-tailed *t*-test, except for panel **C**, in which data were compared using a repeated-measure two-way ANOVA with multiple comparisons. (^*^p < 0.05; ^**^p < 0.01; ^***^p < 0.001; ^****^p < 0.0001).

Given that cell motility pathways were downregulated in *KPV*^−/−^ cells, we assessed whether cell-intrinsic motility properties required vimentin. Cell migration was evaluated using a scratch wound-healing assay. Within 6 h, *KPV*^+/+^ cells had closed ~76% more of the wound area than *KPV*^−/−^ cells (72.16 ± 14.67% vs. 17.71 ± 13.47%, Fig. 3A). To test the invasive potential of *KPV*^+/+^ and *KPV*^−/−^ cells, a Matrigel-coated transwell assay was used to mimic invasion across the alveolar basement membrane. *KPV*^+/+^ cells had a 16-fold increased rate of invasion compared to *KPV*^−/−^ cells (invasive index, 230 ± 41.76 vs. 14.58 ± 2.68, respectively, Fig. 3B). Three-dimensional invasion and migration were modeled by generating *KPV*^+/+^ and *KPV*^−/−^ spheroids. After 48 h of culture in collagen, *KPV*^+/+^ spheroids were 4.65 times larger than *KPV*^−/−^ spheroids, suggesting that, in a three-dimensional model, *KPV*^−/−^ cells have impaired migration and invasion (Fig. 3C).

Withaferin A (WFA) is a steroidal lactone used as an anticancer agent in various murine tumor models, including breast, prostate, and ovarian cancer [34**–**36]. WFA treatment leads to the phosphorylation of vimentin at serine 38 (Ser38) and serine 56 (Ser56), resulting in the aggregation of vimentin intermediate filaments (IFs) [37]. To test the hypothesis that WFA disrupts vimentin IFs and impairs migration and invasion in vitro, we treated *KPV*^+/+^ cells and human lung adenocarcinoma (A549) cells with WFA (Fig. 3D and Supplementary Fig. S3A). In untreated cells, vimentin IFs were extended to the plasma membrane from the nucleus. In WFA-treated cells, vimentin IFs were retracted from the plasma membrane and collapsed around the nucleus (Fig. 3D and Supplementary Fig. S3A). There was no change in vimentin protein levels in cells treated with WFA (Supplementary Fig. S3B) [38]. WFA treatment decreased *KPV*^+/+^ cell migration by ~46% (Fig. 3E) and completely suppressed cell invasion (Fig. 3F). We observed a similar trend in human-derived A549 cells, which exhibited a dose-dependent decrease in cell migration following treatment with WFA (Supplementary Fig. S3C). These data suggest that an intact vimentin intermediate filament network is required for migration and invasion in an in vitro model of NSCLC.

### Withaferin A treatment attenuates cancer progression

**Fig. 4:**
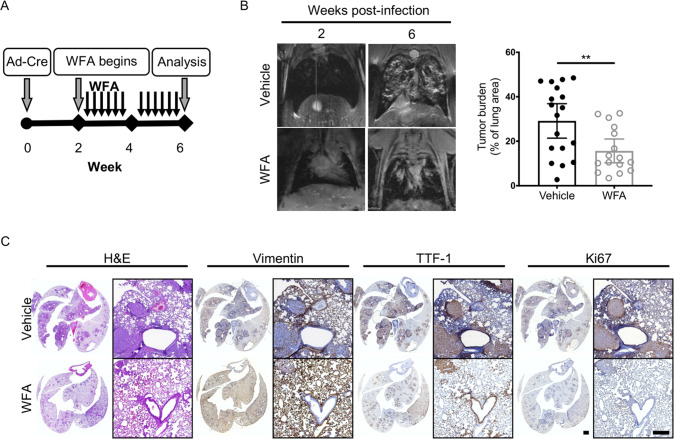
WFA treatment attenuates lung cancer progression. **A** Schematic of experimental design. *KPV*^+/+^ mice were treated with withaferin A (WFA; 4 mg/kg; QOD, p.o.) or vehicle control (DMSO) at 2 weeks post-infection with 10^7^ PFUs of adenoviral Cre. **B** Representative MRI scans show WFA-treated *KPV*^+/+^ lung tumors at 6 weeks post-infection with 10^7^ PFUs of adenoviral Cre (*left*). Dot plot illustrates the tumor volume between WFA-treated or vehicle-treated control *KPV*^+/+^ mice (*right*). Each point represents, for one mouse, the percentage of lung area on MRI occupied by tumor, as measured using Jim software. Data are presented as the mean ± standard deviation (^**^p < 0.01 by unpaired, two-tailed *t*-test). **C** Lungs isolated from vehicle- or WFA-treated KPV^+/+^ mice 6 weeks after adenoviral Cre infection were fixed, sectioned, and subjected to H&E staining and vimentin, TTF-1, and Ki67 immunohistochemical staining. Positively immunostained cells appear brown, and nuclei are dyed blue. Scale bars: 2 mm (whole lungs, *left*), 200 µM (*right*).

To determine whether WFA-mediated disruption of vimentin IF impairs LUAD progression, *KPV*^+/+^ mice were administered WFA (4 mg/kg, QOD, p.o.) 2 weeks after Ad-Cre treatment to initiate tumor development (Fig. 4A). At 6 w.p.i., WFA-treated *KPV*^+/+^ mice developed smaller tumors (tumor burden, 15.65 ± 2.5%) than vehicle-treated mice (tumor burden, 25.1 ± 3.8%) (Fig. 4B). Lung sections were immunostained for vimentin, TTF-1, and Ki67 (Fig. 4C, Supplementary Fig. S4A). Lung tumors from vehicle-treated *KPV*^+/+^ mice had enhanced TTF-1 and Ki67 expression. In contrast, WFA-treated mice had reduced tumor burden with diminished TTF-1 and Ki67 expression (Fig. 4C, Supplementary Fig. S4A). Additional lung tissue sections were stained with pSer56-vimentin antibody to assess the in vivo efficacy of WFA-mediated vimentin disassembly [39] (Supplementary Fig. S4B). Collectively, these data suggest that vimentin can be pharmacologically targeted in vivo to disrupt the ability of lung cancer cells to invade and migrate away from the primary tumor.

### Vimentin confers cancer cell protection from ferroptosis

Cystine/glutamate transporter is an antiporter encoded by the *Slc7a11* and *Slc3a2* genes. This transporter brings cystine into the cell, where it is immediately converted to cysteine, the rate-limiting precursor of the antioxidant glutathione. KRAS-mutant lung adenocarcinoma cells are susceptible to SLC7A11 inhibitor-induced cell death, resulting in attenuated tumor growth in vivo [23]. *KPV*^−/−^ cells express higher levels of *Slc7a11* and *Slc3a2* than *KPV*^+/+^ cells. We reasoned that increased activity of this transporter might lead to higher levels of glutathione in *KPV*^−/−^ cells. Indeed, we identified genes involved in glutathione production, including *Slc1a5*, a glutamine transporter; *Glul*, the enzyme involved in the conversion of glutamine to glutamate; *Shmt1*, an enzyme required for the metabolism of serine to glycine; and *Gclm* and *Gclc*, enzymes involved in the conversion of glutamate to glutathione (Supplementary Fig. S5A).

**Fig. 5:**
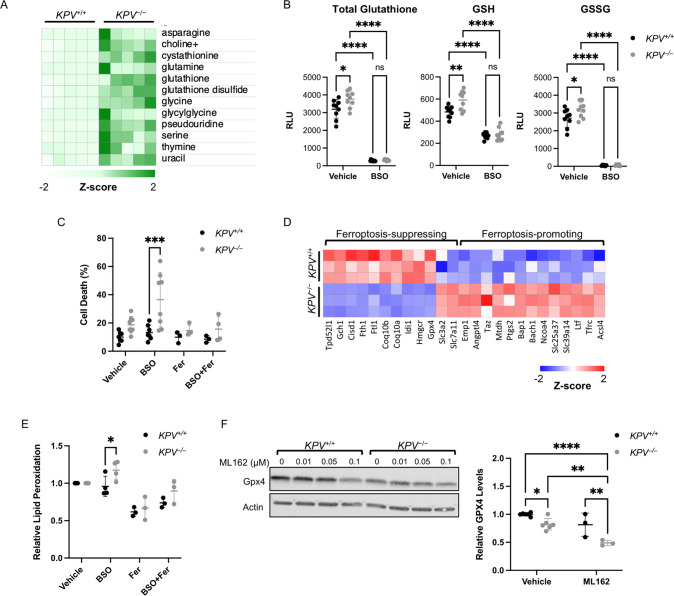
*KPV*^−/−^ cells are susceptible to ferroptosis. **A** Differentially produced metabolites are plotted with each row representing z-scores for each metabolite. Metabolite data were log-transformed and then subjected to an unpaired two-tailed *t*-test; *p*-values were corrected for multiple comparisons (all comparisons shown have adjusted *p*-value < 0.05). **B** Total glutathione and GSSG were quantified in *KPV*^+/+^ and *KPV*^−/−^ cells treated with a vehicle control or BSO for 24 h using a luminescence-based assay (RLU = relative light units). GSH levels were calculated by subtracting GSSG from total glutathione. **C** Cells were treated with indicated reagents for 48 h. Cell death was measured through flow cytometry (*n* = 6–8). **D** Z-scores representing gene expression data from RNA-sequencing. All gene comparisons (*KPV*^+/+^ vs. *KPV*^−/−^) have FDR < 0.05 after adjusting for multiple comparisons; therefore, all gene differences shown between *KPV*^+/+^ vs. *KPV*^−/−^ cells are statistically significant. **E** BODIPY was measured by flow cytometry and normalized to the average vehicle values per experiment (*n* = 6–8). **F** GPX4 levels were measured by Western blot and normalized to an actin loading control and the *KPV*^+/+^ vehicle control. Quantification is from cells treated with 0.1 µM ML162; data were collected from three independent blots (*n* = 3–6). In **B**, **C**, **E**, and **F** groups were compared using a two-way ANOVA with multiple comparisons. All data are presented as the mean ± standard deviation. (^*^p < 0.05; ^**^p < 0.01; ^***^p < 0.001; ^****^p < 0.0001).

Despite expressing similar levels of SLC7A11 protein (Supplementary Fig. S5A), *KPV*^−/−^ cells had much higher levels of glutathione and other metabolites involved in its production than *KPV*^+/+^ cells (Fig. 5A, B, Supplementary Fig. S5C). To test whether *KPV*^−/−^ cells rely on glutathione for survival, we treated the cells with BSO, a well-characterized glutathione-depleting agent [40]. As expected, BSO treatment significantly reduced the glutathione levels compared to cells at baseline and abrogated the difference between *KPV*^+/+^ and *KPV*^−/−^ cells (Fig. 5B). Interestingly, BSO treatment caused elevated cell death in *KPV*^−/−^ cells but did not affect *KPV*^+/+^ cells, suggesting that *KPV*^−/−^ cells rely more on glutathione for survival than *KPV*^+/+^ cells (Fig. 5C).

The accumulation of lipid peroxides triggers ferroptosis. Long-chain fatty acid–CoA ligase 4 (ACSL4) promotes the incorporation of polyunsaturated fatty acids (PUFAs) into phospholipids (PUFA-PLs), which are oxidized into PL-PUFA-OOHs [41, 42]. *Acsl4* is upregulated in *KPV*^−/−^ cells compared to *KPV*^+/+^ cells, suggesting that lipid peroxides may be elevated in *KPV*^−/−^ cells (Fig. 5D). We measured oxidized lipids using C11-BODIPY and found that lipid peroxidation was significantly higher in *KPV*^−/−^ cells than in *KPV*^+/+^ cells under oxidative stress (Fig. 5E). To test whether BSO-mediated cell death in *KPV*^−/−^ cells occurred via ferroptosis, cells were treated with ferrostatin-1 (Fer-1), a drug that blocks lipid peroxidation. Ferrostatin-1 attenuated oxidized lipid levels and reduced the BSO-induced *KPV*^−/−^ cell death (Fig. 5C, E). In agreement with these findings, the ferroptosis biomarker *Ptgs2* is upregulated in *KPV*^−/−^ cells (Fig. 5D) [43]. Together, these results indicate that *KPV*^−/−^ cells have increased lipid peroxidation and ferroptosis under oxidative stress compared to *KPV*^+/+^ cells.

Extrinsic factors and intrinsic factors regulate ferroptosis. Typically, the cystine-glutathione axis is the main extrinsic factor regulating ferroptosis, but because of the seemingly contradictory evidence of increased ferroptosis despite higher levels of the protective antioxidant glutathione in *KPV*^−/−^ cells, we explored other pathways of ferroptosis regulation. Specifically, we assessed whether the intrinsic factor, GPX4 or the extrinsic factor, iron transport, regulates ferroptosis in *KPV*^−/−^ cells. Recently, WFA, which disrupts the vimentin intermediate filament network (Figs. 3 and 4), was shown to induce lipid peroxidation and ferroptosis by suppressing GPX4 activity [44]. GPX4, a key upstream regulator of ferroptosis, is responsible for both converting GSH to glutathione disulfide (GSSG) and reducing phospholipid hydroperoxides (PL-OOHs) to their corresponding alcohol (PL-OHs), thereby preventing the accumulation of lipid peroxides and leading to suppression of ferroptosis. We found that *KPV*^−/−^ cells had reduced *Gpx4* gene expression compared to *KPV*^+/+^ cells (458.0 ± 31.0 vs. 334.2 ± 12.7 CPM, respectively) (Fig. 5D). GPX4 is a selenoperoxidase; its biosynthesis is regulated through the mevalonate pathway [45]. *Hmgcr* and *Idi1* are key components of the mevalonate pathway, giving rise to *Coq10a* and *Coq10b*; these genes are expressed at lower levels in *KPV*^−/−^ cells compared to *KPV*^+/+^ cells (Fig. 5D). GPX4 protein expression, assessed by Western blot on isolated tumor cells and IHC of tumors, was significantly lower in *KPV*^−/−^ cells than in *KPV*^+/+^ cells and in tumors from WFA-treated *KPV*^+/+^ mice compared to vehicle control (Fig. 5F, Supplementary Fig. S5D). Treatment with ML162, a drug that directly targets GPX4, leads to more loss of GPX4 in *KPV*^−/−^ cells compared to *KPV*^+/+^ cells (Fig. 5F). The ML162-mediated decrease of GPX4 protein expression in *KPV*^−/−^ and *KPV*^+/+^ cells was associated with increased cell death, which was prevented by treatment with Fer-1 (Supplementary Fig. 5F). Interestingly, genes that promote iron metabolism such as *Tfrc, Ltf, Slc39a14*, *Slc25a37*, *Ncoa4*, and *Bach1* were upregulated in *KPV*^−/−^ cells, while genes that decrease the cytosolic labile iron pool such as *Ftl*, *Fth1*, and *Cisd1* were downregulated [46**–**49] (Fig. 5D, Supplementary Fig. 5G). Surprisingly, there was no difference in iron levels measured in *KPV*^+/+^ and *KPV*^−/−^ cells (Supplementary Fig. 5E). However, ML162-induced cell death was rescued by the iron chelator deferoxamine (DFO) (Supplementary Fig. 5F), suggesting that iron is required but not sufficient to induce ferroptosis. Together, these results show that several ferroptosis-associated pathways, including loss of intrinsic antioxidant molecules and upregulation of iron transport factors, are working in tandem to induce cell death in *KPV*^−/−^ cells (Supplementary Fig. 5G). Thus, in *KPV*^+/+^ cells, vimentin promotes tumor growth by conferring protection against ferroptosis.

### Vimentin is required for lung cancer metastasis

**Fig. 6:**
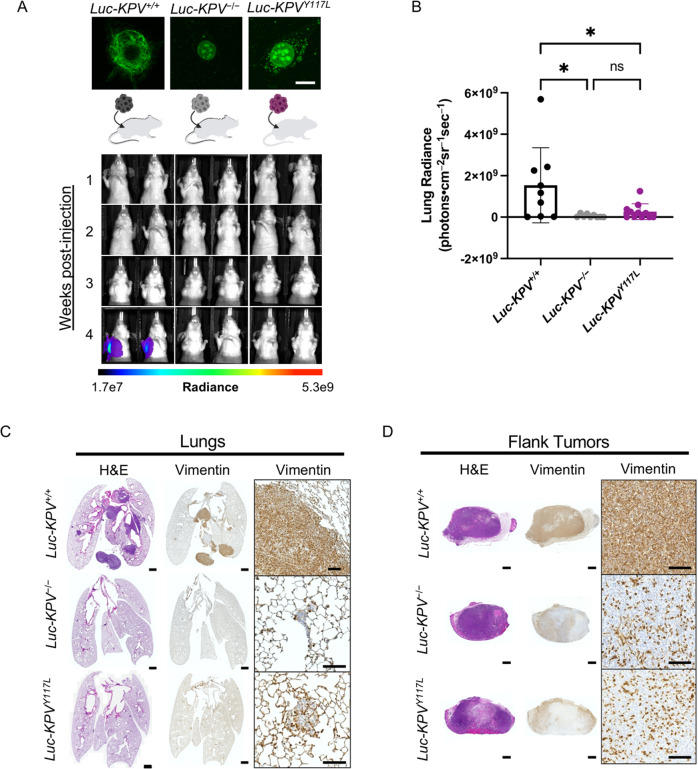
Vimentin is required for accelerated lung cancer metastasis. **A** (*Top*) *Luc*-*KPV*^−/−^ cells were transfected with vimentin-Y117L to create *Luc*-*KPV*^Y117L^ cells. Cells were stained with DAPI and an anti-vimentin antibody (*green*). Scale bar: 10 µm. (*Bottom*) A total of 1 × 10^6^ *KPV*^+/+^, *KPV*^−/−^, or *KPV*^Y117L^ cells labeled with luciferase (*Luc-KPV*^+/+^, *Luc-KPV*^−/−^, and *Luc-KPV*^Y117L^, respectively) were injected subcutaneously into the right flank of nude mice. At 3 weeks post-injection, primary tumors were removed and lung metastases were tracked for an additional 1 week. Shown are representative IVIS images of mice (*n* = 9–11 per group). The coronal views shown were acquired after masking the flank tumor to minimize bleed-through of the signal. Intensity overlay shows the accumulation of luciferase-labeled cells. **B** Luciferin signal was quantified from the lungs. An ordinary one-way ANOVA with multiple comparisons was used to compare groups at week 4 (^*^p < 0.05). **C** Lungs at week 4 and **D** excised flank tumors from week 3 were fixed, sectioned, and subjected to H&E staining and vimentin immunohistochemical staining. Positive vimentin staining is brown, and nuclei are blue. Scale bars: 1 mm (whole tumor/lung, *left*), 100 μm (inset, *right*).

The differences in the growth rate of primary lung tumors in *KPV*^+/+^ and *KPV*^−/−^ mice (see Fig. 1) precluded the ability to determine whether vimentin is required for in vivo metastasis. Therefore, the cell-autonomous ability of vimentin-expressing cells to metastasize was assessed using an allograft tumor model (Fig. 6A). *Luc*-*KPV*^+/+^ cells, a luciferase (*Luc)* expressing cell line that reproducibly colonizes the lung following subcutaneous injection [50, 51], were transfected with CRISPR/Cas9 vimentin knockout plasmid to generate *Luc*-*KPV*^−/−^ cells (Supplementary Fig. S6A). To determine whether an intact vimentin network is required for metastasis, we also generated *Luc*-*KPV*^Y117L^ cells, which express a mutant form of vimentin that cannot form full-length vimentin and instead form punctate unit-length filaments [52] (Fig. 6A). Briefly, nude mice were subcutaneously injected in the right flank with either *Luc*-*KPV*^+/+^, *Luc*-*KPV*^−/−^, or *Luc*-*KPV*^Y117L^ cells (Fig. 6A, Supplementary Fig. S6B). Lung radiance, flank tumor radiance, and flank tumor volume were measured weekly (Fig. 6A, B, Supplementary Fig. S6B–F). At week 4 after injection, mice injected with *Luc*-*KPV*^+/+^ cells had significant lung tumor burdens, as assessed by IVIS imaging and H&E staining (Fig. 6A–C, Supplementary Fig. S6D). In contrast, *Luc*-*KPV*^−/−^and *Luc*-*KPV*^Y117L^ cells failed to form lung tumors. When quantified, the metastatic signal in the lung was significantly higher in *Luc*-*KPV*^+/+^ mice (15.3E8 ± 18.1E8 photons•cm^−2^sr^−1^sec^−1^) than in *Luc*-*KPV*^−/−^ mice (0.564E7 ± 0.754E8 photons•cm^−2^sr^−1^sec^−1^) or *Luc*-*KPV*^Y117L^ mice (2.63E8 ± 3.78E8 photons•cm^−2^sr^−1^sec^−1^). The lungs of *KPV*^+/+^-injected mice displayed large, vimentin-positive metastatic lesions (Fig. 6C). Conversely, the few metastatic tumors formed in the lungs with *KPV*^−/−^ cells were sparse and small; as expected, these tumors did not express vimentin.

While there was no significant difference in flank tumor size between *Luc*-*KPV*^+/+^, *Luc*-*KPV*^−/−^, or *Luc*-*KPV*^Y117L^ conditions at week 1, by week 2, *Luc*-*KPV*^+/+^ flank tumor radiance was higher (23.9E8 ± 12.1E8 vs. 9.10E8 ± 4.62E8 and 8.48E8 ± 3.97E8 photons•cm^−2^sr^−1^sec^−1^, respectively), suggesting that *Luc*-*KPV*^+/+^ tumors grow faster than *Luc*-*KPV*^−/−^ and *Luc*-*KPV*^Y117L^ in vivo (Supplementary Fig. S6C). To remove the effects on metastasis due to differences in proliferation, flank tumors were excised at week 3. The mass of excised *Luc*-*KPV*^+/+^(0.859 ± 0.344 grams) flank tumors was remarkably greater than *Luc*-*KPV*^−/−^ (0.545 ± 0.170 g) and *Luc*-*KPV*^Y117L^ (0.611 ± 0.212 g) tumors (Supplementary Fig. S6F). Excised tumors were stained with H&E and immunostained for vimentin (Fig. 6D). *Luc*-*KPV*^+/+^ cells formed dense tumors that displayed uniform vimentin expression. *Luc*-*KPV*^−/−^ and *Luc*-*KPV*^Y117L^ cells also developed dense tumors; surprisingly, some cells within the tumor expressed vimentin. Based on their spindly or round shapes, we inferred that these cells were infiltrating fibroblasts or macrophages, two cell types that canonically express vimentin. Together, we conclude that vimentin is required for the metastatic spread of murine NSCLC cells. Furthermore, vimentin unit-length filaments are not sufficient to rescue metastasis. Therefore, an intact, mature vimentin intermediate filament network is required for NSCLC metastasis.

## Discussion

Clinically, vimentin expression correlates with increased metastatic potential [18] (Supplementary Fig. S2C), high nuclear grade [53], and poor overall survival across most solid tumor types, including lung, prostate, and breast cancers [2–4]. Vimentin has also been implicated in many aspects of cancer initiation and progression, including tumorigenesis, EMT, and the metastatic spread of cancer [5]. These reports often relied on in vitro experiments comparing cancer cell lines and suppressing vimentin expression with siRNA, shRNA, and pharmacological agents or with cells derived from *Vim*^*−/−*^ mice but lacking oncogene expression. Our data provide causal evidence that vimentin is required for the metastasis of *Kras*-mutant, *Tp53*-null lung cancer cells in vivo and in vitro. Data from the *KPV*^−/−^ GEMM show that vimentin is required for metastasis and tumor progression (Figs. 1 and 6), as *KPV*^−/−^ mice had decreased lung tumor burden (Fig. 1C, Supplementary Fig. S1H), lower-grade tumors (Supplementary Fig. S1K), and no metastasis from primary tumors in the flank to the lung (Fig. 6A–C). Consistent with the decreased metastatic rates, we observed a survival advantage in the *KPV*^−/−^ mice (Fig. 1B). These results were recapitulated in *KPV*^+/+^ mice by disrupting vimentin filaments with WFA treatment two weeks after tumor initiation (Fig. 4). Collectively, these data provide evidence that vimentin is integral in the progression and metastasis of lung cancer.

EMT is the canonical mechanism by which cancer cells lose their epithelial morphology, form invadopodia, and degrade the surrounding basement membrane to promote the invasive spread of cancer [5, 11]. Vimentin expression is upregulated in epithelial cells undergoing EMT in malignant tumors, and several studies support the notion that vimentin functions as a positive regulator of EMT [5, 7, 54–56]. In this respect, it has been proposed that vimentin intermediate filaments provide a scaffold for the recruitment of transcription factors, such as Twist1 and Slug [7]. Specifically, Twist1 upregulates Cullin2 circular RNA, which absorbs vimentin-targeting miRNAs, and thus increases vimentin expression [10]. Accordingly, we found that *KPV*^−/−^ cells lack expression of *Twist1* (Fig. 2). Similarly, when transforming growth factor β (TGF-β) is used to activate Smad-mediated EMT in primary alveolar epithelial cells, the cells undergo a rapid induction of vimentin expression regulated by a Smad-binding-element located in the 5′ promotor region of the *Vim* gene [57]. We used RNA-seq to show that *KPV*^−/−^ cells derived from primary lung tumors display a distinct transcriptional phenotype, characterized by the suppression of genes directly involved in EMT (Fig. 2). Collectively, these data suggest that vimentin should no longer be considered a biomarker of EMT but rather a required protein for transcriptional activation and interaction with transcription factors such Twist and Slug, which serve to induce EMT in epithelial cells.

After a tumor cell has undergone EMT, it will invade the surrounding tissue. We previously reported that the transient expression of vimentin in epithelial cells, which typically express type I and type II keratin intermediate filaments, causes epithelial cells to be transformed into mesenchymal cells, which is accompanied by changes in cell shape, increased cell motility, and focal adhesion dynamics [9, 57]. We use four different methods to show that vimentin is required for cancer cell invasion across the basement membrane and through the interstitial matrix. Direct evidence supporting the role of vimentin in the migration of *Kras*-mutant, *Tp53*-null lung cancer cells was demonstrated by disrupting vimentin expression genetically (Fig. 3A) and pharmacologically in mouse- (Fig. 3E) and human-derived (Supplementary Fig. S3C) cancer cells resulting in impaired migration. Furthermore, loss of vimentin through genetic manipulation (Fig. 3B) and pharmacological interference with WFA (Fig. 3F) confers an inability of cells to invade a layer of the basement membrane. *KPV*^−/−^ cells also fail to invade through a collagen-rich matrix in a three-dimensional experimental model (Fig. 3C). Importantly, we provide evidence that vimentin is required for in vivo metastasis. In the *KPV*^+/+^ mice, we observed metastases in the liver at 12 weeks after tumor induction; *KPV*^−/−^ mice had no evidence of metastatic lesions (Supplementary Fig. S1D). To confirm this observation, we implanted cells in the flanks of mice. *KPV*^+/+^ cells, but not *KPV*^−/−^ cells nor *KPV*^Y117L^ cells, were able to invade and migrate away from the primary tumor and seed metastatic lesions in the lung (Fig. 6). Together, these results provide direct evidence that an intact vimentin intermediate filament network is required for the cell motility that leads to metastasis.

To our knowledge, this is the first report to show that vimentin protects cancer cells from ferroptosis (Fig. 5). Specifically, we show that *KPV*^−/−^ cells are more susceptible to BSO-induced death than *KPV*^+/+^ cells, and that this effect can be rescued with Fer-1. Importantly, we show that several convergent mechanisms of ferroptosis are activated in *KPV*^−/−^, but not *KPV*^+/+^, cells (Supplementary Fig. S5F). These include loss of the intrinsic mechanism of GPX4-mediated protection from lipid peroxidation and elevation of the extrinsic mechanism of iron transport. Interestingly, previous studies support our findings that suggest vimentin may interact with GPX4, be involved in iron metabolism, and play a role in lipid droplet (LD) maintenance. A report from Hassannia et al. recently showed that WFA treatment directly targets vimentin also binds and inactivates GPX4, thus inducing ferroptosis [44]. In line with this finding, we show that GPX4 levels are lower in *KPV*^−/−^ cells compared to *KPV*^+/+^ cells (Fig. 5D, F). Additionally, suppressors of the redox pathway upstream of GPX4 (glutathione, SLC7A11) are increased or unchanged in *KPV*^−/−^ cells (Fig. 5A, B, D, Supplementary Fig. S5A–C). In contrast, downstream readouts such as lipid peroxidation and cell death are decreased (Fig. 5C–E), suggesting that there may be a direct relationship between GPX4 and vimentin. Several iron-related genes, including the transferrin receptor (*Trfc*), *Ltf*, *Slc39a14*, *Slc25a37*, *Ncoa4*, and *Bach1,* are upregulated in *KPV*^−/−^ cells (Fig. 5D). In contrast, genes associated with negative regulation of the cytosolic iron pool, such as *Ftl*, *Fth1*, and *Cisd1,* are downregulated in *KPV*^−/−^ cells. However, there is no difference in iron levels between *KPV*^+/+^ and *KPV*^−/−^ cells (Supplementary Fig. S5E) and no difference between groups after treatment with the iron chelator DFO alone or with ML162 (Supplementary Fig. S5F). Interestingly, vimentin knockdown regulatory T cells (Tregs) express higher transferrin receptor levels and take up more fatty acids than wildtype Tregs [58]. However, the precise role of vimentin in iron metabolism remains to be explored. Degradation of LDs promotes ferroptosis [59]. Interestingly, vimentin forms a cage around LDs and physically anchors LDs via the linker protein perilipin [60]. Vimentin-null cells have smaller LDs, impaired lipid motility, and impaired lipolysis [61, 62]. In support of the hypothesis that differences in lipolysis might lead to susceptibility to ferroptosis in *KPV*^−/−^ cells, we observed that *Gch1*, which is the rate-limiting enzyme of BH_4_-mediated lipid remodeling, and *Tpd52l1*, which promotes lipid storage, are upregulated in *KPV*^+/+^ cells compared to *KPV*^−/−^ cells [59, 63] (Fig. 5D). These data suggest that the interaction between vimentin and lipid droplets may mediate ferroptosis. Of note, many of the studies mentioned in this section were conducted in non-cancer cell types, and the precise role of vimentin in regulating ferroptosis in KRAS-mutant cancer remains to be identified.

Global vimentin depletion confers a survival advantage in the *Kras*-mutant, *Tp53*-null lung cancer model. Additionally, by treating *KPV*^+/+^ mice with WFA following tumor development, we show that the delayed tumor growth and metastasis observed when vimentin is suppressed is not due solely to delayed onset of tumor growth, but rather to attenuated growth kinetics in tumor cells that lack an intact vimentin network. Although vimentin-null mice were first reported to display no obvious phenotype, these data along with previous reports suggest that loss of vimentin is protective against a range of disease states including lung cancer, acute lung injury, acute respiratory distress syndrome, idiopathic pulmonary fibrosis, bacterial meningitis, cerebral ischemia, and acute colitis [26, 64–68]. These global vimentin knockout mice lack vimentin expression in tumor cells and tumor microenvironment (TME). The TME, which includes immune and stromal cells, can promote metastasis of primary tumor cells. The cytokine interleukin 1β (IL-1β), produced by macrophages in the lung, is a mediator of cancer growth, metastasis, and tumor-associated macrophage infiltration [65, 69]. Our group has reported that vimentin is required for mature IL-1β release [65]. Because of the potential contribution of the TME in vimentin-null mice, we set out to ensure that vimentin is sufficient in cancer cells to promote metastasis. We injected *KPV*^+/+^ or *KPV*^−/−^ cells into wildtype nude mice; these mice lack T cells but retain innate immune cells. We observed recruited mesenchymal and immune cells in subcutaneous flank tumors (Fig. 6D), suggesting that the vimentin-positive TME in this model participates in growth of the primary tumor. Despite being in the presence of vimentin-expressing stromal and immune cells, *KPV*^−/−^ cells failed to metastasize. Therefore, while other groups have found that vimentin deficiency impairs function in cancer-associated fibroblasts and immune cells such as macrophages and T cells [56, 58, 65, 70], a vimentin-expressing microenvironment is not sufficient to promote metastasis in the time frame evaluated. To better understand how vimentin participates in different compartments of the TME, we recognize that animal models with immune-, mesenchymal-, and epithelial-specific deletion of vimentin will need to be created.

This study fills a major gap in the literature by providing novel causal data that vimentin is required for the in vivo progression of NSCLC at several steps of the metastatic cascade. Importantly, our in vivo data validated our in vitro data and in vitro data from previously published reports [11, 12, 71–74]. Additionally, this work gives causal context to clinical data associating vimentin expression with tumor progression in patients [2, 18, 75]. By using in vivo models and disrupting vimentin both genetically and pharmacologically, we have presented physiological context to these reports. Through this study, we have identified vimentin as a new clinical target for metastatic lung cancer.

## Materials and methods

### Murine lung cancer model

All animal experiments were approved by Northwestern University’s Institutional Animal Care and Use Committee (IACUC). Sex-matched 6–10-week-old mice were used for all in vivo experiments. *LSL*-*Kras*^G12D/+^; *Tp53*^flox/flox^ (*KPV*^+/+^) mice were bred as described by DuPage and colleagues and were generously gifted to us by Dr. Navdeep Chandel (Northwestern University, Chicago, IL) [25]. Vimentin knockout mice were a gift from Albee Messing (University of Wisconsin, Madison, WI). Vimentin knockout mice were crossed with *KPV*^+/+^ mice to create *KPV*^−/−^ mice. *KPV*^+/+^, *KPV*^−/−^, and the validated *Rosa26-LSL-LacZ* mice were administered adenovirus expressing Cre recombinase (Ad-Cre; ViraQuest) or a null adenovirus (Ad-Null) via intratracheal instillation (1 × 10^9^ PFUs unless otherwise noted) under isoflurane anesthesia [32]. Survival was monitored daily. Weight was monitored weekly.

### Magnetic resonance imaging

Scheduled magnetic resonance imaging (MRI) was performed at Northwestern University Center for Translational Imaging (Chicago, IL) via a 7-tesla system (Clinscan, Bruker) using a four-channel mouse body coil at set time points (2, 6, and 10 weeks after Ad-Cre administration). Mice were anesthetized with isoflurane (2% isoflurane in oxygen for induction, followed by 1.5–2% via nose cone for maintenance during imaging) to permit tolerance to imaging. Pulse oximetry and respiration were recorded and used to trigger the MRI to avoid motion artifacts. Turbo Multi Spin Echo imaging sequence was used in conjunction with respiratory triggering to acquire in vivo MRI coronal images covering all the lung area and portions of abdomen, including liver and kidneys (ST = 0.5 mm, In plane = 120 μm, TR = 1000 msec, TE = 20 msec). Gradient Echo sequence was used with cardiac triggering (pulse oximeter rate) covering the lung area transversally (ST = 0.5 mm, In plane = 120 μm, TR ~ 20 msec, TE ~ 2 msec). Jim software was used to quantify tumor burden (Xinapse).

### Immunohistochemistry

Mice were anesthetized and lungs were perfused via the right ventricle with 4% paraformaldehyde in phosphate-buffered saline (PBS). A 20-gauge angiocatheter was sutured into the trachea, heart and lungs were removed en bloc, and then lungs were inflated with 0.8 mL of 4% paraformaldehyde at a pressure not exceeding 16 cm H_2_O. Tissue was fixed in 4% paraformaldehyde in PBS overnight at 4 °C, then processed, embedded in paraffin, and sectioned (4–5 μm). Sections (5 μm) were cut from three distinct zones (top, middle, bottom) of each paraffin block and were stained with hematoxylin and eosin (H&E) or used for immunohistochemistry. H&E-stained samples were assessed for tumor type (hyperplasia, adenoma, adenocarcinoma), numbers, and areas using AxioVision digital image processing software per manufacturer’s protocol. Briefly, the desired quantification region was imaged, total region area measured (μm^2^), pixels matching tumor cells selected, and a binary mask created to compute tumor numbers and area. The measurements were independently confirmed by manual grading of tumor lesions. Tumor lesions were graded by a pathologist as described previously [76].

For immunohistochemistry, tissue was rehydrated and subjected to antigen retrieval in 10 mM sodium citrate (pH = 6.0) with 0.05% Tween-20 for 20 min at 96–98 °C, followed by 20 min of cooling. Tissue sections were blocked in 3% hydrogen peroxide for 5 min. A Vector Laboratories avidin/biotin blocking kit (SP-2001), Vectastain ABC kit (PK-4001), and 3,3′-diaminobenzidine (DAB) peroxidase substrate kit (SK-4100) were used according to the manufacturer’s protocols. Nuclei were counterstained with hematoxylin (Thermo Scientific 72604) and treated with bluing solution (Thermo Scientific 7301), and then coverslips were mounted with Cytoseal 60 (Thermo Scientific 8310-4). A TissueGnostics automated slide imaging system was used to acquire whole-tissue images and measure area. The semi-quantitative immunohistochemistry (IHC) method was performed using ImageJ Fiji software to conduct deconvolution and downstream analysis as described [77]. Human tissue was purchased from Biomax (LC2083) and stained as described above.

### Cell isolation and culture

*KPV*^+/+^ and *KPV*^−/−^ mice were treated with Ad-Cre as described above; after 6 weeks, mice were sacrificed and lung tumors were excised. The tissue was dissociated into a single-cell suspension in 0.2 mg/mL DNase and 2 mg/mL collagenase D and filtered through a 40 μm filter. Cells then underwent two rounds of selection. First, cells were treated with anti-CD45 magnetic beads (Miltenyi Biotec, 130-052-301) and were passed through a magnetic column. CD45-negative cells were then subjected to anti-EpCAM magnetic beads (Miltenyi Biotec, 130-105-958) and underwent positive selection. CD45-negative, EpCAM-positive cells were expanded in vitro and were used in experiments between passages 1 and 10. Cells derived from a human lung adenocarcinoma (A549) were obtained from the American Type Culture Collection (ATCC, Manassas, VA). All cells were maintained in Dulbecco’s modified Eagle medium (DMEM) supplemented with 10% fetal bovine serum, 100 U/mL penicillin, 100 µg/mL streptomycin, and HEPES buffer. All cells were grown in a humidified incubator of 5% CO_2_/95% air at 37 °C.

### Polymerase chain reaction

Mice were infected with Ad-Null or Ad-Cre; mice were sacrificed at 2, 8, and 12 w.p.i. and lungs were harvested. Lungs were lysed, and DNA was extracted and amplified by polymerase chain reaction (PCR) using the following primers: *Kras* forward, GGC CTG CTG AAA ATG ACT GAG TAT A; *Kras* reverse, CTG TAT CGT CAA GGC GCT CTT; *Kras-G12D* forward, CTTGTGGTGGTTGGAGCTGA; and *Kras-G12D* reverse, TCCAAGAGACAGGTTTCTCCA. DNA products were run on an agarose gel and imaged with the Li-Cor Odyssey imaging system.

### Western blotting

Western blot analysis was utilized to quantify protein levels in cell lysates. The protein was separated using 12% sodium dodecyl sulfate polyacrylamide gel electrophoresis (SDS-PAGE) and transferred onto nitrocellulose membranes. Membranes were then blocked with Odyssey blocking buffer (Li-Cor Biosciences) and incubated with the appropriate primary antibodies overnight at 4 °C. IRDye secondary antibodies were then used (Li-Cor Biosciences, 1:10,000) for 2 h at room temperature. Images of blots were acquired using the Li-Cor Odyssey Fc Imaging System.

### RNA-sequencing

Tumor cells were isolated from *KPV*^+/+^ and *KPV*^−/−^ mice at 6 w.p.i. and underwent CD45-negative, EpCAM-positive magnetic-activated cell sorting (MACS) selection as described above. Cells were cultured for one passage, then lysed using RLT lysis buffer (Qiagen), and total RNA was isolated with the RNeasy Plus Mini Kit (Qiagen). RNA quality was confirmed with a TapeStation 4200 (Agilent); all samples had an RNA integrity number (RIN) score equal to or greater than 9.8. Next, mRNA was isolated via poly(A) enrichment (NEBNext). Libraries were prepared using NEBNext RNA Ultra chemistry (New England Biolabs). Sequencing was performed on an Illumina NextSeq 500 using a 75-cycle single-end high-output sequencing kit. Reads were demultiplexed (bcl2fastq), and fastq files were aligned to the mm10 mouse reference genome with TopHat2. Htseq was used to obtain counts. The resulting data were filtered, and differentially expressed genes (DEGs) were identified using the edgeR package. DEGs were selected using a false discovery rate (FDR) cutoff of <0.05, with a 1.0-fold change cutoff for pairwise comparison. K-means clustering and heat map visualization was performed using the Morpheus web tool (https://software.broadinstitute.org/morpheus). Enrichment analysis was performed using Gorilla [78, 79]. The RNA-seq data have been deposited in NCBI’s Gene Expression Omnibus and are accessible through GEO Series accession number GSE225083.

### Withaferin A treatments

Withaferin A (WFA) was purchased from Enzo Life Sciences and dissolved in dimethyl sulfoxide (DMSO; Sigma-Aldrich) to a final concentration of 5 μM unless noted otherwise. For in vivo experiments, jelly pellets were utilized to provide an oral, voluntary method of drug delivery. Using a 24-well flat-bottom tissue culture plate as the jelly mold, WFA (4 mg/kg in DMSO) or vehicle control (DMSO only) were combined with gelatin and Splenda for flavoring as described elsewhere [80]. Tumor development was induced with Ad-Cre as described above. Two weeks following Ad-Cre administration, mice were fed jelly pellets every other day for 4 weeks. Survival was tracked daily and weight was measured weekly.

### Scratch wound assay

Cells were grown to 100% confluence in 6-well plates. A pipette tip was used to make a single scratch in the monolayer. The cells were washed with 1× PBS to remove debris and imaged at 0 and 6 h. For WFA conditions, WFA or DMSO was added at 0 h (when the scratch was created). Rate of cell migration was calculated using ImageJ software. Results were normalized to the initial wound area at 0 h.

### Matrigel invasion assay

Transwell inserts with 8 μm pores were coated with Matrigel (200 μg/mL), and 5 × 10^4^ *KPV*^+/+^ or *KPV*^−/−^ cells were seeded atop each transwell in serum-free media. For WFA experiments, cells were resuspended in WFA or DMSO containing media directly before being seeded in transwells. Media containing 10% fetal bovine serum was added to the bottom well to serve as a chemoattractant. Cells were placed at 37 °C for 48 h. Following incubation, the Matrigel with the cells remaining on the upper surface of the transwell was removed with a cotton swab. The cells remaining on the bottom of the membrane were fixed in 2% paraformaldehyde and incubated with Hoechst nuclear dye (Invitrogen; 1:10,000 in 1× PBS). Five random 10× magnification fields were imaged, and the average number of cells per field was quantified; this average is reported as the “invasive index.”

### Spheroid culture

Spheroids were generated as described by Gilbert-Ross et al. [81]. Briefly, cells were grown in Nunclon Sphera 96-well plates (Thermo-Fisher Scientific) at a concentration of 3000 cells per well. After 3 days in culture, cells were transferred using a wide-bore pipette tip to 2 mg/mL collagen (Corning) in 4-well LabTek plates (Nunc). Collagen was allowed to gel at 37 °C for 1 h; then, complete media was added to the spheroids. Gels were imaged using a Ti2 widefield microscope (Nikon) at 0, 24, and 48 h. Spheroid area was quantified using Fiji software. Reported spheroid area values are normalized to 0-h spheroid area of the same spheroid.

### Metabolomics

*KPV*^+/+^ and *KPV*^−/−^ cells were grown in 6-well plates. High-performance liquid chromatography (HPLC) grade methanol (80% in water) was added to cells, and plates were incubated at −80 °C for 20 min. Lysates were collected and centrifuged, and the supernatant was collected and analyzed by high-performance liquid chromatography and high-resolution mass spectrometry and tandem mass spectrometry (HPLC-MS/MS). Specifically, the system consisted of a Thermo Q-Exactive in line with an electrospray source and an Ultimate3000 (Thermo) series HPLC consisting of a binary pump, degasser, and auto-sampler outfitted with a Xbridge Amide column (Waters; dimensions of 4.6 mm × 100 mm and a 3.5 µm particle size). The mobile phase A contained 95% (vol/vol) water, 5% (vol/vol) acetonitrile, 20 mM ammonium hydroxide, 20 mM ammonium acetate, pH = 9.0; B was 100% acetonitrile. The gradient was as follows: 0 min, 15% A; 2.5 min, 30% A; 7 min, 43% A; 16 min, 62% A; 16.1–18 min, 75% A; 18–25 min, 15% A with a flow rate of 400 μL/min. The capillary of the ESI source was set to 275 °C, with sheath gas at 45 arbitrary units, auxiliary gas at 5 arbitrary units, and the spray voltage at 4.0 kV. In positive/negative polarity switching mode, an *m*/*z* scan range from 70 to 850 was chosen and MS1 data were collected at a resolution of 70,000. The automatic gain control (AGC) target was set at 1 × 10^6^ and the maximum injection time was 200 ms. The top 5 precursor ions were subsequently fragmented, in a data-dependent manner, using the higher energy collisional dissociation (HCD) cell set to 30% normalized collision energy in MS2 at a resolution power of 17,500. Besides matching *m/z*, metabolites were identified by matching retention time with analytical standards and/or MS2 fragmentation pattern. Data acquisition and analysis were performed using Xcalibur 4.1 software and Tracefinder 4.1 software, respectively (both from Thermo Fisher Scientific). For each sample, peak area of each metabolite was normalized to total ion count per sample. Data were log-transformed and compared with a two-tailed, unpaired *t*-test. Data were analyzed with MetaboAnalyst software [82].

### Preparation of cells for subcutaneous flank injection

*KPV*^+/+^ cells labeled with luciferase (*Luc*-*KPV*^+/+^ cells) were a generous gift from Dr. Navdeep Chandel. To create *Luc*-*KPV*^−/−^ cells, *Luc*-*KPV*^+/+^ cells were transfected with a commercially available CRISPR/Cas9 vimentin knockout plasmid according to manufacturer’s directions (Santa Cruz Biotechnology sc-423676). *Luc*-*KPV*^Y117L^ cells were created as previously described [52].

### Tracking of tumor growth in subcutaneous flank injection model

Male nude (NU/J) mice were purchased from Jackson Laboratories; 8–12-week-old mice were anesthetized with 2% isoflurane in oxygen and were given a subcutaneous injection of cells (1 × 10^6^ cells in 100 μL of 1× PBS) on their right flanks. Weight and tumor volume were monitored weekly. For IVIS imaging, mice were injected with 150 mg of D-luciferin per kilogram of body weight (PerkinElmer 770504). After 10 min, IVIS images were captured. At week 3 post-injection, tumors were removed. Briefly, mice were anesthetized with ketamine (100 mg/kg body weight) and xylazine (10 mg/kg body weight). Tumor area was disinfected with 70% ethanol and iodide solution. Tumors were excised and placed in 4% paraformaldehyde for immunohistochemistry. Wounds were closed with simple interrupted nylon sutures (Ethilon). Mice were monitored until they recovered from anesthesia; they were housed singly and treated with Meloxicam as an analgesic. The following week, mice underwent a final IVIS imaging session and were sacrificed.

### Immunofluorescence confocal microscopy

Cells were grown on no. 1 glass coverslips for all immunofluorescent immunocytochemistry experiments. Following treatment, *KPV*^+/+^ and *KPV*^−/−^ cells were fixed in methanol for 3–5 min. A549 cells were fixed with 2% paraformaldehyde for 7–10 min. *KPV*^+/+^ and *KPV*^−/−^ cells were blocked in 5% normal goat serum (NGS) for 1 h at room temperature. A549 cells were blocked with 1.5% NGS for 30 min at 37 °C. Cells were then treated with the indicated primary antibodies overnight at 4 °C. Cells were washed twice in PBS with 0.10% Tween-20 for 3 min each and treated with secondary antibodies conjugated with Alexa Fluor 488 (Invitrogen A-11039, 1:200) and/or Alexa Fluor 568 (Invitrogen A-11004, 1:200), as well as Hoechst nuclear dye (Invitrogen H3570, 1:10,000). Coverslips were mounted and sealed. A Nikon A1R+ laser scanning confocal microscope equipped with ×60 and ×100 objective lenses was used to acquire images. For experiments with A549 cells, a Zeiss LSM 510 laser scanning confocal microscope equipped with a ×63 objective lens was used to acquire images. Nikon NIS-Elements software and ImageJ were used for image processing.

### Flow cytometry

Lipid peroxidation was assessed by flow cytometry with BODIPY™ 581/591 C11 (Thermo Fisher Scientific, United States) following the manufacturer’s instructions. In brief, cells were pretreated with 1 µM BSO or 20 µM Fer-1. Cells were collected at 48 h for lipid peroxidation and to measure cell viability. Cells were resuspended in 400 μl of serum-free medium that contained BODIPY™ 581/591 C11 (5 µM) at 37 °C in the dark for 30 min, to detect lipid peroxidation. Finally, cells were stained with Ghost Dye Red 780 (Tonbo Biosciences) and analyzed by flow cytometry immediately. FeRhoNox (Gorya Chemical) was used following manufacturer’s instructions. Briefly, cells were plated overnight and incubated with FeRhoNox in HBSS for 1 h then immediately analyzed by flow cytometry. Data were collected using a BD FACSymphony cell analyzer, and a minimum of 10,000 cells were analyzed per condition. Cells were gated to exclude debris and doublets using FlowJo software and statistical analysis was conducted as described below.

### Statistical analysis

Statistical analyses were performed in GraphPad Prism v.9 software using statistical tests indicated in the figure legends. Data are presented as mean ± s.d. with a minimum of *n* = 3 independent experiments unless otherwise indicated. Specific numbers of replicates are indicated in scatter plots. Experiments were neither randomized nor blinded, excluding histological analysis. Differences between two groups were assessed by using a student’s *t*-test. Differences between three or more groups were assessed using one-way analysis of variance (ANOVA) with a Bonferroni multiple comparisons test. Values of *p* < 0.05 were significant. The log rank test, which calculates the chi-square (χ^2^) for each event time for each group and sums the results, was used in the Kaplan–Meier curve analysis. Minimum sample group sizes were determined through power analysis. Specific tests are indicated in figure legends. Plated cells were allocated randomly to each treatment group; mice were assigned randomly to each treatment group.

### Reagents

All antibodies used are summarized in Supplementary Table 1.

## Supplementary information

Supplemental Figure Legends

Supp Figure 1

Supp Figure 2

Supp Figure 3

Supp Figure 4

Supp Figure 5

Supp Figure 6

Supp Table 1

## References

[1] Siegel RL, Miller KD, Jemal A (2019). Cancer statistics, 2019. CA Cancer J Clin.

[2] Dauphin M, Barbe C, Lemaire S, Nawrocki-Raby B, Lagonotte E, Delepine G (2013). Vimentin expression predicts the occurrence of metastases in non small cell lung carcinomas. Lung Cancer.

[3] Burch TC, Watson MT, Nyalwidhe JO (2013). Variable metastatic potentials correlate with differential plectin and vimentin expression in syngeneic androgen independent prostate cancer cells. PLoS One.

[4] Domagala W, Lasota J, Dukowicz A, Markiewski M, Striker G, Weber K (1990). Vimentin expression appears to be associated with poor prognosis in node-negative ductal NOS breast carcinomas. Am J Pathol.

[5] Kidd ME, Shumaker DK, Ridge KM (2014). The role of vimentin intermediate filaments in the progression of lung cancer. Am J Respir Cell Mol Biol.

[6] Dongre A, Weinberg RA (2019). New insights into the mechanisms of epithelial-mesenchymal transition and implications for cancer. Nat Rev Mol Cell Biol.

[7] Virtakoivu R, Mai A, Mattila E, De Franceschi N, Imanishi SY, Corthals G (2015). Vimentin-ERK Signaling Uncouples Slug Gene Regulatory Function. Cancer Res.

[8] Francart ME, Vanwynsberghe AM, Lambert J, Bourcy M, Genna A, Ancel J (2020). Vimentin prevents a miR-dependent negative regulation of tissue factor mRNA during epithelial-mesenchymal transitions and facilitates early metastasis. Oncogene.

[9] Mendez MG, Kojima S, Goldman RD (2010). Vimentin induces changes in cell shape, motility, and adhesion during the epithelial to mesenchymal transition. FASEB J.

[10] Meng J, Chen S, Han JX, Qian B, Wang XR, Zhong WL (2018). Twist1 Regulates Vimentin through Cul2 Circular RNA to Promote EMT in Hepatocellular Carcinoma. Cancer Res.

[11] Schoumacher M, Goldman RD, Louvard D, Vignjevic DMActin (2010). microtubules, and vimentin intermediate filaments cooperate for elongation of invadopodia. J Cell Biol.

[12] Helfand BT, Mendez MG, Murthy SN, Shumaker DK, Grin B, Mahammad S (2011). Vimentin organization modulates the formation of lamellipodia. Mol Biol Cell.

[13] Hendrix MJ, Seftor EA, Seftor RE, Trevor KT (1997). Experimental co-expression of vimentin and keratin intermediate filaments in human breast cancer cells results in phenotypic interconversion and increased invasive behavior. Am J Pathol.

[14] Gilles C, Polette M, Zahm JM, Tournier JM, Volders L, Foidart JM (1999). Vimentin contributes to human mammary epithelial cell migration. J Cell Sci.

[15] Messica Y, Laser-Azogui A, Volberg T, Elisha Y, Lysakovskaia K, Eils R (2017). The role of Vimentin in Regulating Cell Invasive Migration in Dense Cultures of Breast Carcinoma Cells. Nano Lett.

[16] Wang W, Yi M, Zhang R, Li J, Chen S, Cai J (2018). Vimentin is a crucial target for anti-metastasis therapy of nasopharyngeal carcinoma. Mol Cell Biochem.

[17] Chan SH, Tsai JP, Shen CJ, Liao YH, Chen BK (2017). Oleate-induced PTX3 promotes head and neck squamous cell carcinoma metastasis through the up-regulation of vimentin. Oncotarget.

[18] Liu S, Liu L, Ye W, Ye D, Wang T, Guo W (2016). High Vimentin Expression Associated with Lymph Node Metastasis and Predicated a Poor Prognosis in Oral Squamous Cell Carcinoma. Sci Rep.

[19] Zelenko Z, Gallagher EJ, Tobin-Hess A, Belardi V, Rostoker R, Blank J (2017). Silencing vimentin expression decreases pulmonary metastases in a pre-diabetic mouse model of mammary tumor progression. Oncogene.

[20] Zhu QS, Rosenblatt K, Huang KL, Lahat G, Brobey R, Bolshakov S (2011). Vimentin is a novel AKT1 target mediating motility and invasion. Oncogene.

[21] Yang CY, Chang PW, Hsu WH, Chang HC, Chen CL, Lai CC (2019). Src and SHP2 coordinately regulate the dynamics and organization of vimentin filaments during cell migration. Oncogene.

[22] Tang D, Chen X, Kang R, Kroemer G (2021). Ferroptosis: molecular mechanisms and health implications. Cell Res.

[23] Hu K, Li K, Lv J, Feng J, Chen J, Wu H (2020). Suppression of the SLC7A11/glutathione axis causes synthetic lethality in KRAS-mutant lung adenocarcinoma. J Clin Invest.

[24] Ni J, Chen K, Zhang J, Zhang X (2021). Inhibition of GPX4 or mTOR overcomes resistance to Lapatinib via promoting ferroptosis in NSCLC cells. Biochem Biophys Res Commun.

[25] DuPage M, Dooley AL, Jacks T (2009). Conditional mouse lung cancer models using adenoviral or lentiviral delivery of Cre recombinase. Nat Protoc.

[26] Colucci-Guyon E, Portier MM, Dunia I, Paulin D, Pournin S, Babinet C (1994). Mice lacking vimentin develop and reproduce without an obvious phenotype. Cell.

[27] Shajani-Yi Z, de Abreu FB, Peterson JD, Tsongalis GJ (2018). Frequency of Somatic TP53 Mutations in Combination with Known Pathogenic Mutations in Colon Adenocarcinoma, Non-Small Cell Lung Carcinoma, and Gliomas as Identified by Next-Generation Sequencing. Neoplasia.

[28] Scoccianti C, Vesin A, Martel G, Olivier M, Brambilla E, Timsit JF (2012). Prognostic value of TP53, KRAS and EGFR mutations in nonsmall cell lung cancer: the EUELC cohort. Eur Respir J.

[29] Cox AD, Fesik SW, Kimmelman AC, Luo J, Der CJ (2014). Drugging the undruggable RAS: Mission possible?. Nat Rev Drug Disco.

[30] Cancer Genome Atlas Research N. (2014). Comprehensive molecular profiling of lung adenocarcinoma. Nature.

[31] Jackson EL, Olive KP, Tuveson DA, Bronson R, Crowley D, Brown M (2005). The differential effects of mutant p53 alleles on advanced murine lung cancer. Cancer Res.

[32] Soriano P (1999). Generalized lacZ expression with the ROSA26 Cre reporter strain. Nat Genet.

[33] Whithaus K, Fukuoka J, Prihoda TJ, Jagirdar J (2012). Evaluation of napsin A, cytokeratin 5/6, p63, and thyroid transcription factor 1 in adenocarcinoma versus squamous cell carcinoma of the lung. Arch Pathol Lab Med.

[34] Nagalingam A, Kuppusamy P, Singh SV, Sharma D, Saxena NK (2014). Mechanistic elucidation of the antitumor properties of withaferin a in breast cancer. Cancer Res.

[35] Suman S, Das TP, Moselhy J, Pal D, Kolluru V, Alatassi H (2016). Oral administration of withaferin A inhibits carcinogenesis of prostate in TRAMP model. Oncotarget.

[36] Kakar SS, Parte S, Carter K, Joshua IG, Worth C, Rameshwar P (2017). Withaferin A (WFA) inhibits tumor growth and metastasis by targeting ovarian cancer stem cells. Oncotarget.

[37] Bargagna-Mohan P, Hamza A, Kim YE (2007). Khuan Abby Ho Y, Mor-Vaknin N, Wendschlag N et al. The tumor inhibitor and antiangiogenic agent withaferin A targets the intermediate filament protein vimentin. Chem Biol.

[38] Bollong MJ, Pietila M, Pearson AD, Sarkar TR, Ahmad I, Soundararajan R (2017). A vimentin binding small molecule leads to mitotic disruption in mesenchymal cancers. Proc Natl Acad Sci USA.

[39] Thaiparambil JT, Bender L, Ganesh T, Kline E, Patel P, Liu Y (2011). Withaferin A inhibits breast cancer invasion and metastasis at sub-cytotoxic doses by inducing vimentin disassembly and serine 56 phosphorylation. Int J Cancer.

[40] Griffith OW (1982). Mechanism of action, metabolism, and toxicity of buthionine sulfoximine and its higher homologs, potent inhibitors of glutathione synthesis. J Biol Chem.

[41] Doll S, Proneth B, Tyurina YY, Panzilius E, Kobayashi S, Ingold I (2017). ACSL4 dictates ferroptosis sensitivity by shaping cellular lipid composition. Nat Chem Biol.

[42] Dixon SJ, Winter GE, Musavi LS, Lee ED, Snijder B, Rebsamen M (2015). Human Haploid Cell Genetics Reveals Roles for Lipid Metabolism Genes in Nonapoptotic Cell Death. ACS Chem Biol.

[43] Yang WS, SriRamaratnam R, Welsch ME, Shimada K, Skouta R, Viswanathan VS (2014). Regulation of ferroptotic cancer cell death by GPX4. Cell.

[44] Hassannia B, Wiernicki B, Ingold I, Qu F, Van Herck S, Tyurina YY (2018). Nano-targeted induction of dual ferroptotic mechanisms eradicates high-risk neuroblastoma. J Clin Invest.

[45] Friedmann Angeli JP, Conrad M (2018). Selenium and GPX4, a vital symbiosis. Free Radic Biol Med.

[46] Gao M, Monian P, Quadri N, Ramasamy R, (2015). Jiang X. Glutaminolysis and Transferrin Regulate Ferroptosis. Mol Cell.

[47] Yu Y, Jiang L, Wang H, Shen Z, Cheng Q, Zhang P (2020). Hepatic transferrin plays a role in systemic iron homeostasis and liver ferroptosis. Blood.

[48] Yuan H, Li X, Zhang X, Kang R, Tang D (2016). CISD1 inhibits ferroptosis by protection against mitochondrial lipid peroxidation. Biochem Biophys Res Commun.

[49] Wang Y, Liu Y, Liu J, Kang R, Tang D (2020). NEDD4L-mediated LTF protein degradation limits ferroptosis. Biochem Biophys Res Commun.

[50] Romero R, Sayin VI, Davidson SM, Bauer MR, Singh SX, LeBoeuf SE (2017). Keap1 loss promotes Kras-driven lung cancer and results in dependence on glutaminolysis. Nat Med.

[51] Gibbons DL, Lin W, Creighton CJ, Rizvi ZH, Gregory PA, Goodall GJ (2009). Contextual extracellular cues promote tumor cell EMT and metastasis by regulating miR-200 family expression. Genes Dev.

[52] Robert A, Rossow MJ, Hookway C, Adam SA, Gelfand VI (2015). Vimentin filament precursors exchange subunits in an ATP-dependent manner. Proc Natl Acad Sci USA.

[53] Thomas PA, Kirschmann DA, Cerhan JR, Folberg R, Seftor EA, Sellers TA (1999). Association between keratin and vimentin expression, malignant phenotype, and survival in postmenopausal breast cancer patients. Clin Cancer Res.

[54] Peuhu E, Virtakoivu R, Mai A, Warri A, Ivaska J (2017). Epithelial vimentin plays a functional role in mammary gland development. Development.

[55] Wang Z, Divanyan A, Jourd’heuil FL, Goldman RD, Ridge KM, Jourd’heuil D (2018). Vimentin expression is required for the development of EMT-related renal fibrosis following unilateral ureteral obstruction in mice. Am J Physiol Ren Physiol.

[56] Cheng F, Shen Y, Mohanasundaram P, Lindstrom M, Ivaska J, Ny T (2016). Vimentin coordinates fibroblast proliferation and keratinocyte differentiation in wound healing via TGF-beta-Slug signaling. Proc Natl Acad Sci USA.

[57] Rogel MR, Soni PN, Troken JR, Sitikov A, Trejo HE, Ridge KM (2011). Vimentin is sufficient and required for wound repair and remodeling in alveolar epithelial cells. FASEB J.

[58] McDonald-Hyman C, Muller JT, Loschi M, Thangavelu G, Saha A, Kumari S (2018). The vimentin intermediate filament network restrains regulatory T cell suppression of graft-versus-host disease. J Clin Invest.

[59] Bai Y, Meng L, Han L, Jia Y, Zhao Y, Gao H (2019). Lipid storage and lipophagy regulates ferroptosis. Biochem Biophys Res Commun.

[60] Heid H, Rickelt S, Zimbelmann R, Winter S, Schumacher H, Dorflinger Y (2014). On the formation of lipid droplets in human adipocytes: the organization of the perilipin-vimentin cortex. PLoS One.

[61] Shen WJ, Patel S, Eriksson JE, Kraemer FB (2010). Vimentin is a functional partner of hormone sensitive lipase and facilitates lipolysis. J Proteome Res.

[62] Shen WJ, Zaidi SK, Patel S, Cortez Y, Ueno M, Azhar R (2012). Ablation of vimentin results in defective steroidogenesis. Endocrinology.

[63] Kraft VAN, Bezjian CT, Pfeiffer S, Ringelstetter L, Muller C, Zandkarimi F (2020). GTP Cyclohydrolase 1/Tetrahydrobiopterin Counteract Ferroptosis through Lipid Remodeling. ACS Cent Sci.

[64] Mor-Vaknin N, Legendre M, Yu Y, Serezani CH, Garg SK, Jatzek A (2013). Murine colitis is mediated by vimentin. Sci Rep..

[65] dos Santos G, Rogel MR, Baker MA, Troken JR, Urich D, Morales-Nebreda L (2015). Vimentin regulates activation of the NLRP3 inflammasome. Nat Commun.

[66] Huang SH, Chi F, Peng L, Bo T, Zhang B, Liu LQ (2016). Vimentin, a Novel NF-kappaB Regulator, Is Required for Meningitic Escherichia coli K1-Induced Pathogen Invasion and PMN Transmigration across the Blood-Brain Barrier. PLoS One.

[67] Jiang SX, Slinn J, Aylsworth A, Hou ST (2012). Vimentin participates in microglia activation and neurotoxicity in cerebral ischemia. J Neurochem.

[68] Surolia R, Li FJ, Wang Z, Li H, Dsouza K, Thomas V (2019). Vimentin intermediate filament assembly regulates fibroblast invasion in fibrogenic lung injury. JCI Insight.

[69] Karki R, Kanneganti TD (2019). Diverging inflammasome signals in tumorigenesis and potential targeting. Nat Rev Cancer.

[70] Richardson AM, Havel LS, Koyen AE, Konen JM, Shupe J, Wiles WG (2018). Vimentin Is Required for Lung Adenocarcinoma Metastasis via Heterotypic Tumor Cell-Cancer-Associated Fibroblast Interactions during Collective Invasion. Clin Cancer Res.

[71] Lanier MH, Kim T, Cooper JA (2015). CARMIL2 is a novel molecular connection between vimentin and actin essential for cell migration and invadopodia formation. Mol Biol Cell.

[72] Challa AA, Stefanovic B (2011). A novel role of vimentin filaments: binding and stabilization of collagen mRNAs. Mol Cell Biol.

[73] Gan Z, Ding L, Burckhardt CJ, Lowery J, Zaritsky A, Sitterley K (2016). Vimentin Intermediate Filaments Template Microtubule Networks to Enhance Persistence in Cell Polarity and Directed Migration. Cell Syst.

[74] Costigliola N, Ding L, Burckhardt CJ, Han SJ, Gutierrez E, Mota A (2017). Vimentin fibers orient traction stress. Proc Natl Acad Sci USA.

[75] Al-Saad S, Al-Shibli K, Donnem T, Persson M, Bremnes RM, Busund LT (2008). The prognostic impact of NF-kappaB p105, vimentin, E-cadherin and Par6 expression in epithelial and stromal compartment in non-small-cell lung cancer. Br J Cancer.

[76] Nikitin AY, Alcaraz A, Anver MR, Bronson RT, Cardiff RD, Dixon D (2004). Classification of proliferative pulmonary lesions of the mouse: recommendations of the mouse models of human cancers consortium. Cancer Res.

[77] Crowe AR, Yue W (2019). Semi-quantitative Determination of Protein Expression using Immunohistochemistry Staining and Analysis: An Integrated Protocol. Bio Protoc.

[78] Eden E, Lipson D, Yogev S, Yakhini Z (2007). Discovering motifs in ranked lists of DNA sequences. PLoS Comput Biol.

[79] Eden E, Navon R, Steinfeld I, Lipson D, Yakhini Z (2009). GOrilla: a tool for discovery and visualization of enriched GO terms in ranked gene lists. BMC Bioinforma.

[80] Zhang L, Lee NJ, Nguyen AD, Enriquez RF, Riepler SJ, Stehrer B (2010). Additive actions of the cannabinoid and neuropeptide Y systems on adiposity and lipid oxidation. Diabetes Obes Metab.

[81] Gilbert-Ross M, Konen J, Koo J, Shupe J, Robinson BS, Wiles WG (2017). Targeting adhesion signaling in KRAS, LKB1 mutant lung adenocarcinoma. JCI Insight.

[82] Chong J, Soufan O, Li C, Caraus I, Li S, Bourque G (2018). MetaboAnalyst 4.0: towards more transparent and integrative metabolomics analysis. NuclAcids Res.

