# Supplementary material for: Vimentin is required for tumor progression and metastasis in a mouse model of non–small cell lung cancer
Source: Oncogene. 2023 May 9;42(25):2074–87. doi: 10.1038/s41388-023-02703-9 (PMC10275760; doi:10.1038/s41388-023-02703-9)
Supplement: Supplementary file 1 — Supplemental Figure Legends [file 41388_2023_2703_MOESM1_ESM.pdf]

Vimentin is Required for Tumor Progression and Metastasis in a Mouse Model of Non-Small Cell Lung Cancer

Alexandra L. Berr[1,2], Kristin Wiese[2], Gimena dos Santos[2], Clarissa M. Koch[2], Kishore R. Anekalla[2], Martha Kidd[2], Jennifer M. Davis[2], Yuan Cheng[2], Yuan-Shih Hu[2], Karen M. Ridge[2,3]

[1]Department of Biomedical Engineering, Northwestern University, Chicago, IL, USA
[2]Division of Pulmonary and Critical Care Medicine, Northwestern University, Chicago, IL, USA
[3]Department of Cell and Molecular Biology, Northwestern University, Chicago, IL, USA

The authors have no conflicts of interest to declare.

Supported by: AB and KW were supported by NIH/NHLBI T32 HL076139; KRA was supported by David and Christina Cugell Fellowship; KMR was supported by NIH P01HL071643, R01HL128194, P01AG049665.

Corresponding Author:

Karen M. Ridge, PhD
Northwestern University
Division of Pulmonary and Critical Care
Department of Cell and Molecular Biology
303 E. Superior Avenue
SQBRC 5-520
Chicago, IL 60611
kridge@northwestern.edu

Supplemental Figure Legends

Supplemental Figure 1. (**A**) Experimental design. *LSL-Kras*$^{G12D/+}$*Tp53*$^{flox/flox}$ (*KPV*$^{+/+}$) mice were crossed with vimentin-knockout (*Vim*$^{-/-}$) mice. *KPV*$^{+/+}$ and *KPV*$^{-/-}$ mice were administered adenoviral Cre recombinase (Ad-Cre) which resulted in gene recombination at LoxP sites. As a control, null adenovirus (Ad-null) was administered to an independent cohort of mice. (**B**) Lungs were isolated from Ad-null-treated *KPV*$^{+/+}$ and *KPV*$^{-/-}$ mice, fixed, sectioned, and subjected to H&E staining and vimentin immunohistochemical staining. Positive vimentin staining is brown, and nuclei are blue. Scale bar: 200 μm. (**C**) *Rosa26-LSL-LacZ* reporter mice were administered Ad-Null or Ad-Cre, and β-galactosidase staining was performed on whole lung samples; positive staining appears blue. (**D**) *KPV*$^{+/+}$ and *KPV*$^{-/-}$ mice were administered Ad-Null or Ad-Cre. Lungs were harvested at 2, 8, and 12 weeks following adenoviral infection. DNA was isolated from the tissue and PCR was performed to evaluate the presence of the wild-type (WT) and mutant (G12D) *Kras* transcript. (**E**) Tumor cells were isolated from Ad-Cre-infected mice at 6 weeks post-infection. A Western blot was performed on *KPV*$^{+/+}$ and *KPV*$^{-/-}$ whole cell lysates to detect WT and G12D-mutant KRAS. P53 mRNA (**F**) and protein levels (**G**) were detected through qRT-PCR and Western blot, respectively; MLE-12 cells were used as a positive control. (**H**) *KPV*$^{+/+}$ and *KPV*$^{-/-}$ mice were infected with null or Cre recombinase adenovirus (Ad-Null and Ad-Cre, respectively) and were imaged at 2, 6, and 10 weeks post-infection (w.p.i.). Representative MRI coronal (*left*) and transverse (*right*) images are shown. Hearts (H) are outlined in white. (**I**) Positive staining was quantified from lung sections stained with either TTF-1 or Ki67 (see **Figure 1D** for representative images) and normalized to either total lung area (TTF-1) or total tumor cell count (Ki67) (n=2-5). (**J**) *KPV*$^{+/+}$ and *KPV*$^{-/-}$ cells were plated overnight and were then treated with BrdU for 4 hours. BrdU incorporation was detected and normalized to the *KPV*$^{+/+}$ condition for each independent trial (n=4). Data were compared using an unpaired, two-tailed t-test (**p<0.01). (**K**) H&E-stained lung sections from 8 or 12 w.p.i. were evaluated for tumor grade by a pathologist.

(**J**) Human lung tissue sections (or lymph node tissue where indicated) were stained with antibodies against vimentin. LUAD=lung adenocarcinoma. Positive vimentin staining is brown, and nuclei are blue. Scale bar: 200 µm. Data are presented as the mean ± standard deviation.

Supplemental Figure 2. (**A**) $KPV^{+/+}$ and $KPV^{-/-}$ cell lysates were subjected to a Western blot to detect E-cadherin, N-cadherin, vimentin, and actin. Band signal was quantified and normalized to actin loading controls and average $KPV^{+/+}$ signal. Data were compared using an unpaired, two-tailed t-test (*p<0.01; ***p<0.001; ****p<0.0001). (**B**) $KPV^{+/+}$ and $KPV^{-/-}$ cells were stained for vimentin (*green*), for keratin 8 (*red*), and with Hoechst nuclear dye (*blue*). Scale bar: 10 µm. (**C-E**) Messenger RNA from $KPV^{+/+}$ and $KPV^{-/-}$ cell lysates was quantified via RNA sequencing. (**C**) Principal component analysis (PCA) plot with each point representing one replicate (*black,* $KPV^{+/+}$; *grey,* $KPV^{-/-}$). (**D**) Pearson's correlation plot. The correlation coefficient for each comparison is shown. (**E**) MA plot. Genes of interest are indicated. (**F**) Lungs were harvested from $KPV^{+/+}$ and $KPV^{-/-}$ mice at 7 w.p.i., fixed, sectioned, and subjected to immunohistochemistry with antibodies against E-cadherin and N-cadherin. Scale bar: 1 mm (whole lung), 100 µm (insets). Representative images and quantification of positive staining are shown (n=2-5).

Supplemental Figure 3. (**A**) A549 cells were treated with 2 µM withaferin A (WFA) for 1 hour. Cells were fixed and stained for vimentin (white). A phase contrast image was used to identify cell borders (dashed line). Scale bar: 10 µm, 5 µm *(inset*). (**B**) A549 cells were treated with WFA for the indicated dose and time. A Western blot is shown; vimentin and GAPDH antibodies were used to probe for these proteins. (**C**) A549 cells were treated with DMSO control or 1 or 2 µM WFA and subjected to a scratch wound assay. After 24 hours, wound closure was assessed. Representative images (*left*) and quantitation (*right*) are shown. Data were compared to the vehicle control using an unpaired, two-tailed t-test (*p<0.05; ***p<0.001).

Supplemental Figure 4. (**A**) Lungs isolated from vehicle- or WFA-treated $KPV^{+/+}$ mice at 6 weeks after adenoviral Cre infection were fixed, sectioned, and subjected to immunohistochemistry for the indicated markers; slides were scanned and signal was quantified using Histoquest. TTF-1 and Ki67 were normalized to total lung area. Data were subject to an unpaired, two-tailed t-test (**p<0.01; ***p<0.001). (**B**) Lungs isolated from vehicle- and WFA-treated $KPV^{+/+}$ mice were sectioned and stained for phospho-Serine55 vimentin (green) and DAPI. Scale bar: 50 µm, 5 µm (*insets*).

Supplemental Figure 5. (**A**) Select gene expression values from RNA sequencing. All gene comparisons shown ($KPV^{+/+}$ vs. $KPV^{-/-}$) have FDR<0.05 after adjusting for multiple comparisons; therefore, all gene differences shown between $KPV^{+/+}$ vs. $KPV^{-/-}$ cells are statistically significant. (**B**) SLC7A11 levels were measured by Western blot and normalized to an actin loading control and the average $KPV^{+/+}$ control. (**C**) Metabolite data were normalized to the total ion count per sample, log-transformed, and subjected to an unpaired, two-tailed t-test. P-values were corrected for multiple comparisons (*adjusted p-value<0.05). (**D**) Lungs were harvested from $KPV^{+/+}$ mice treated with WFA or vehicle control at 6 weeks after adenoviral Cre infection, fixed, sectioned, and subjected to anti-GPX antibody staining. Scale bar: 1 mm. (**E**) $KPV^{+/+}$ and $KPV^{-/-}$ cells were treated with the ML162 (1 µM), DFO (100 µM), and/or Fer-1 (10 µM) for 48 hours; cell death was quantified with an LDH assay. Groups were compared using a 2-way ANOVA with multiple comparisons. All data are presented as the mean ± standard deviation. (****p<0.0001). (**F**) A schematic model representing the ferroptosis-related mechanisms that are affected by loss of vimentin in KRAS-mutant, p53-null lung adenocarcinoma cells. Created with BioRender.com

Supplemental Figure 6. (**A**) Luciferase-tagged $KPV^{+/+}$ (*Luc*-$KPV^{+/+}$) cells were treated with CRISPR-Cas9 to knock out vimentin (*Luc*-$KPV^{-/-}$). Cells were subjected to a Western blot and probed for vimentin and actin. (**B**) Representative IVIS images for each week after flank injection

(n=9-11 mice per group). At 3 weeks post-injection, primary tumors were removed and lung metastases were tracked for an additional 1 week. Intensity overlays show the accumulation of luciferase-labeled cells. Luciferin signal was quantified from primary flank tumors (**C**) and the lungs (**D**). (**F**) Flank tumor volume was measured with calipers each week. Volume was calculated using the formula Volume=(length^2 x width)/2. For **C**-**E**, data were subjected to a two-way ANOVA with multiple comparisons. (**G**) At week 3, tumors were removed and weighed. For **G**, data were subjected to a one-way ANOVA with multiple comparisons. (*p<0.05; **p<0.01). Data are presented as the mean ± standard deviation.

Supplemental Table 1. List of antibodies used. For proteins with multiple antibodies used, the figures in which they are used are indicated. If no figure is indicated, the antibody was used for all instances in which that protein was detected. **WB**=Western blot; **IHC**=immunohistochemistry; **IF**=immunofluorescence.
