# Supplementary figures and images for: Vimentin is required for tumor progression and metastasis in a mouse model of non–small cell lung cancer
Source: Oncogene. 2023 May 9;42(25):2074–87. doi: 10.1038/s41388-023-02703-9 (PMC10275760; doi:10.1038/s41388-023-02703-9)

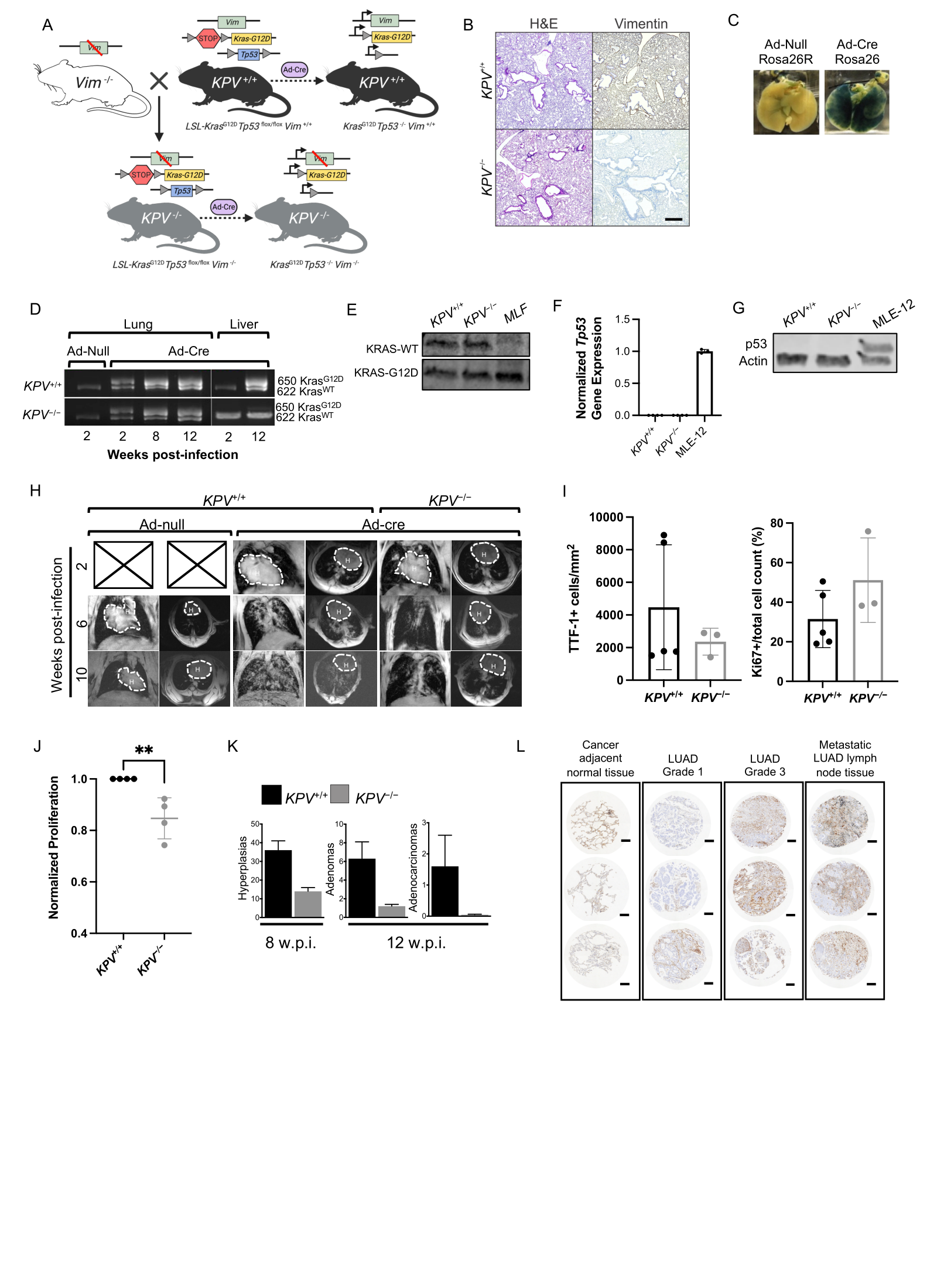

Supplement: Supplementary file 2 — Supp Figure 1 [file 41388_2023_2703_MOESM2_ESM.tif]

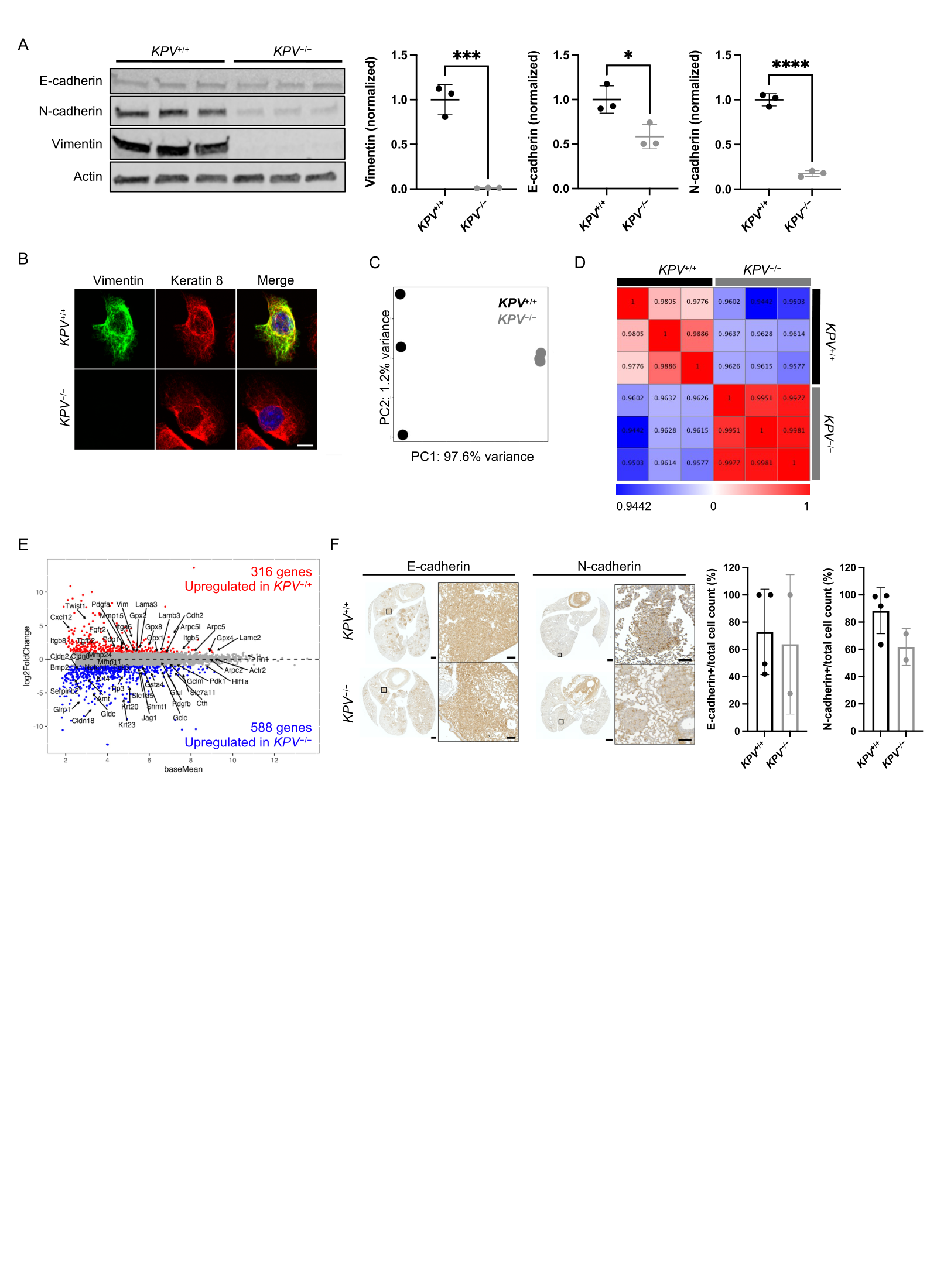

Supplement: Supplementary file 3 — Supp Figure 2 [file 41388_2023_2703_MOESM3_ESM.tif]

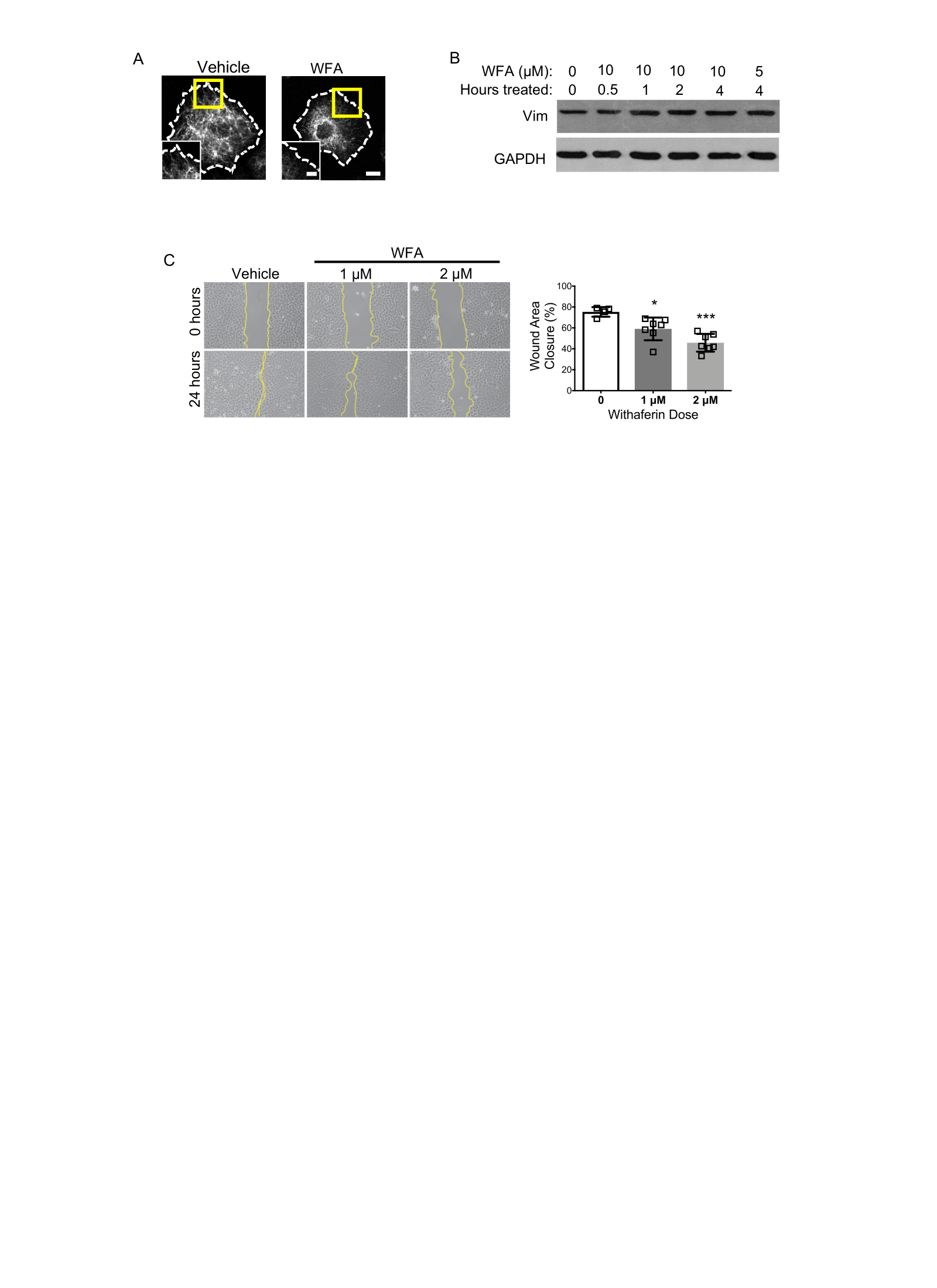

Supplement: Supplementary file 4 — Supp Figure 3 [file 41388_2023_2703_MOESM4_ESM.tif]

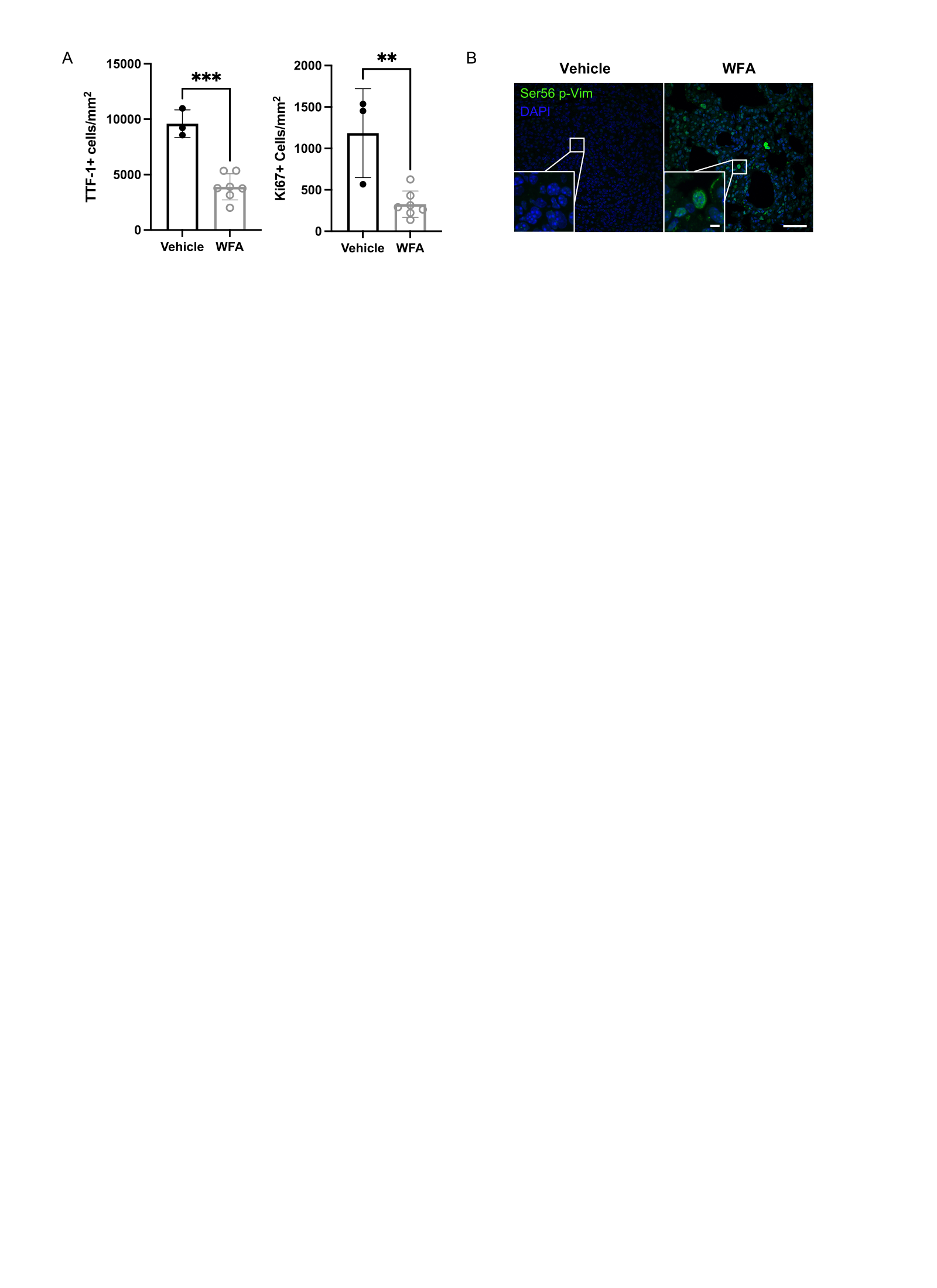

Supplement: Supplementary file 5 — Supp Figure 4 [file 41388_2023_2703_MOESM5_ESM.tif]

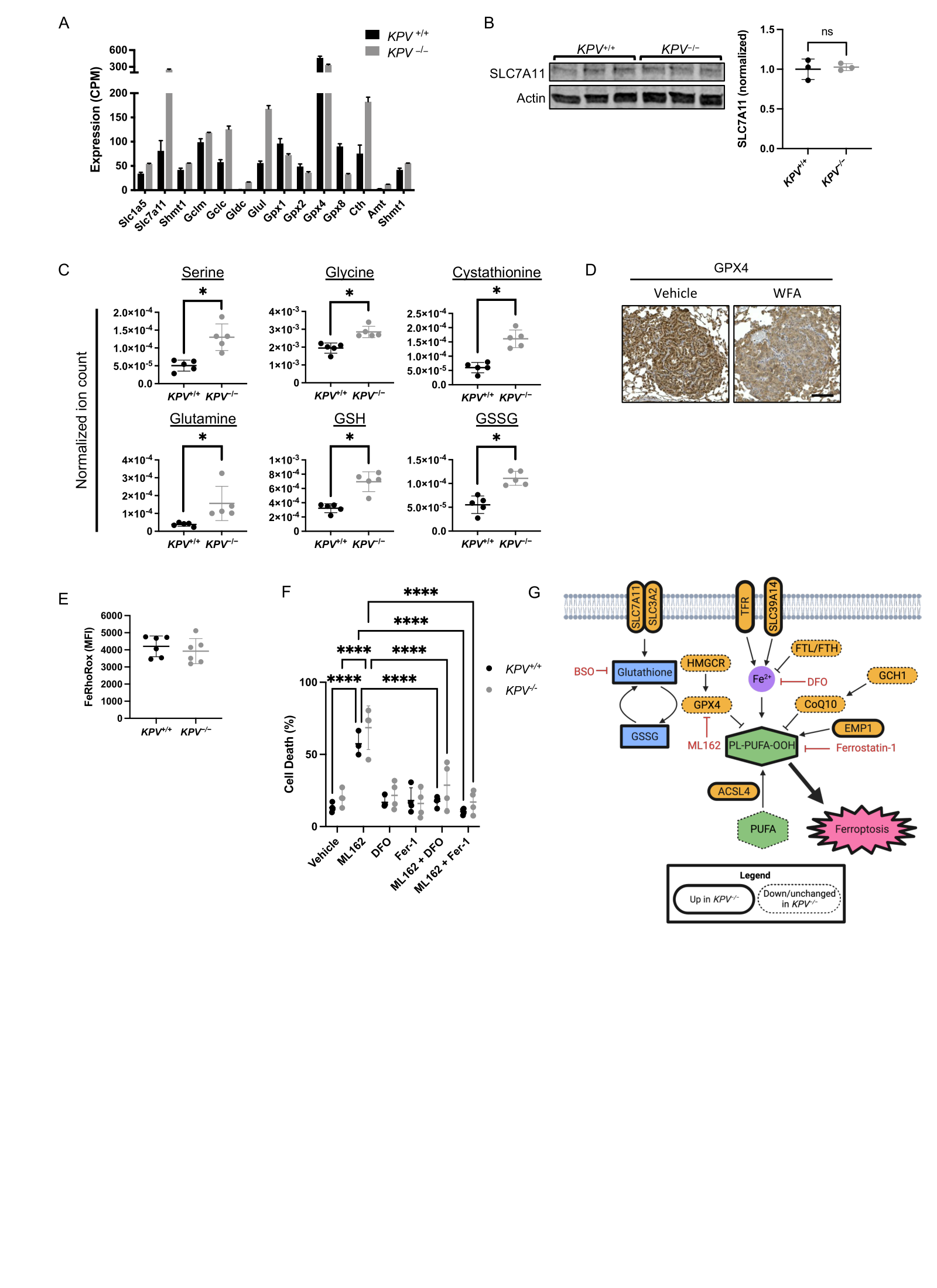

Supplement: Supplementary file 6 — Supp Figure 5 [file 41388_2023_2703_MOESM6_ESM.tif]

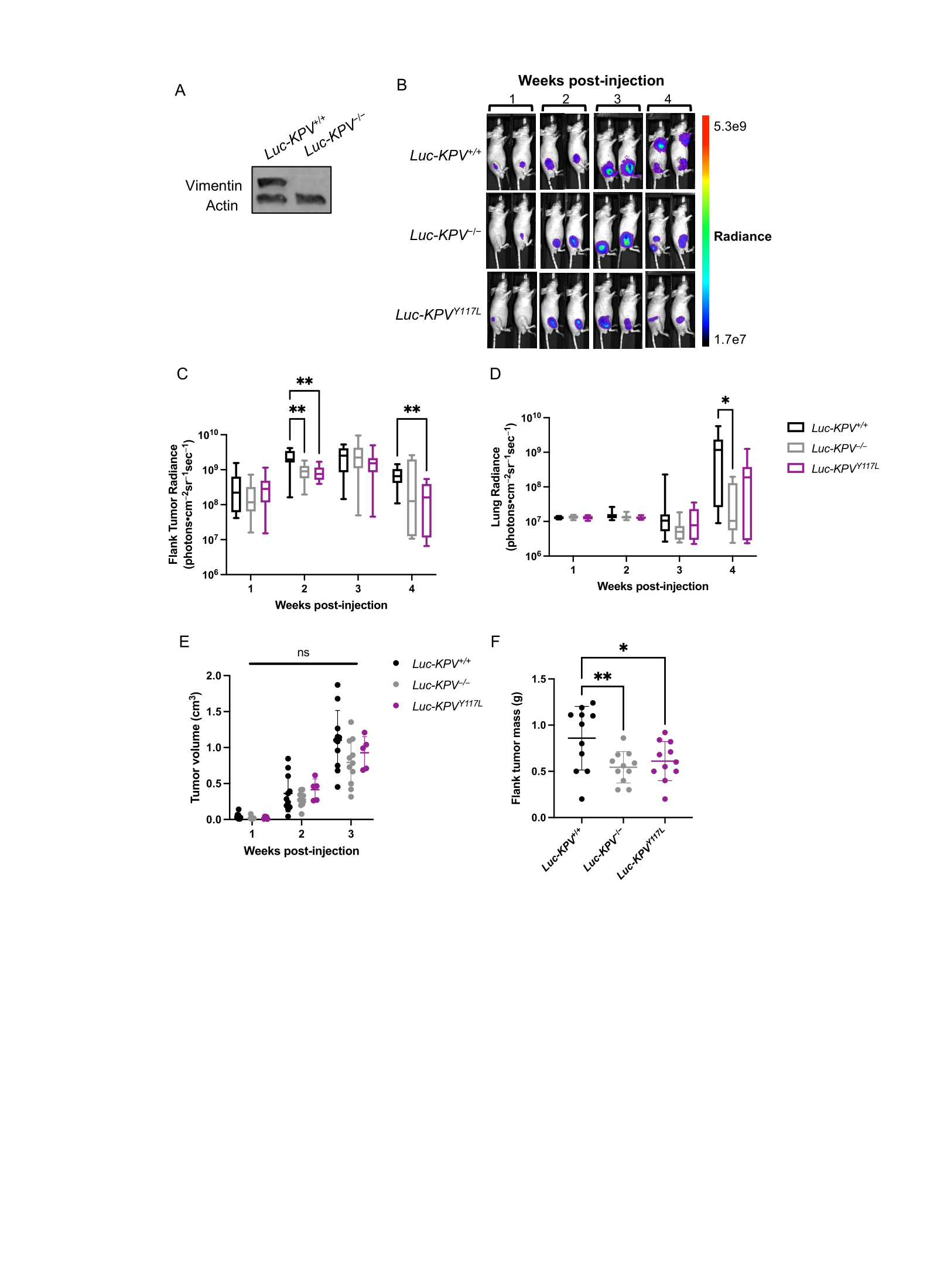

Supplement: Supplementary file 7 — Supp Figure 6 [file 41388_2023_2703_MOESM7_ESM.tif]
